# The Joint Effects of Acoustic and Linguistic Markers for Early Identification of Mild Cognitive Impairment

**DOI:** 10.3389/fdgth.2021.702772

**Published:** 2022-02-11

**Authors:** Fengyi Tang, Jun Chen, Hiroko H. Dodge, Jiayu Zhou

**Affiliations:** ^1^Department of Computer Science of Engineering, Michigan State University, East Lansing, MI, United States; ^2^Department of Bioinformatics, University of Michigan, Ann Arbor, MI, United States; ^3^Department of Neurology, Layton Aging and Alzheimer's Disease Center, Oregon Health & Science University, Portland, OR, United States

**Keywords:** mild cognitive impairment (MCI), Alzheimer's disease, behavioral intervention, audio and linguistic markers, conversations, I-CONECT project

## Abstract

In recent years, behavioral markers such as spoken language and lexical preferences have been studied in the early detection of mild cognitive impairment (MCI) using conversations. While the combination of linguistic and acoustic signals have been shown to be effective in detecting MCI, they have generally been restricted to structured conversations in which the interviewee responds to fixed prompts. In this study, we show that linguistic and acoustic features can be combined synergistically to identify MCI in semi-structured conversations. Using conversational data from an on-going clinical trial (Clinicaltrials.gov: NCT02871921), we find that the combination of linguistic and acoustic features on semi-structured conversations achieves a mean AUC of 82.7, significantly (*p* < 0.01) out-performing linguistic-only (74.9 mean AUC) or acoustic-only (65.0 mean AUC) detections on hold-out data. Additionally, features (linguistic, acoustic and combination) obtained from semi-structured conversations outperform their counterparts obtained from structured weekly conversations in identifying MCI. Some linguistic categories are significantly better at predicting MCI status (e.g., death, home) than others.

## Introduction

Detection of dementia at an early MCI stage has been of great interest in recent years for effective prevention of dementia as well as clinical trials enrichment. However, detecting subtle declines associated with early MCI is difficult. Recently, there has been a growing interest in the use of linguistic-based language and acoustic-based behavioral markers—characteristics related to language use (1), speech (2–4), cognitive capacity (5), and lexical preferences (6) (henceforth referred as behavioral markers in this paper)—in early detection. This is because behavioral markers can provide easy accessibility and are generally more cost-effective (7) to obtain than biological markers such as PET/CT scans (8, 9). Several studies (1, 6) have demonstrated the effectiveness of linguistic markers in early detection using features extracted from short, semi-structured conversations. In semi-structured conversations, there are no set interview questions; instead, conversations are led by the participant themselves.

Although linguistic markers have been shown to work effectively in predicting MCI (1, 6, 10, 11), combining with acoustic markers have also been shown to provide strong predictive capacity for both AD and MCI detection. Although the underlying biological mechanisms for the association between acoustic features and cognition have not been well documented, the brain controls human muscle movement. Cognitive impairment can lead to impairments in neuromuscular motor coordination and is likely to affect acoustic speech outputs including pause rates and speed of speech (12). However, most of the studies focusing on acoustic features are thus far limited to responses to structured conversations (e.g., responses to standardized cognitive tests and fixed questionnaires). It is unknown whether acoustic markers can be used in semi-structured conversations and if combining linguistic and acoustic markers can improve the detection of MCI. Most predictive studies using acoustic markers rely on either fixed prompts (4, 13) or pronunciation tasks (4) to control for the differences in linguistic contexts used in conversations. This is because variations in utterance lengths, word choices and sentence structures can introduce variance in acoustic features independent of vocal differences. In fact, Roark et al. points out that “narrow and topic-focused use of language” is important for “more accurate” acoustic marker extraction (4). However, while these studies suggest that acoustic markers can have very high predictive value, the highly structured conversational settings may restrict the effectiveness of linguistic markers. Traditionally, semi-structured conversational settings have been used in linguistic marker studies because they reflect participant linguistic preferences in open conversations (without fixed sentences). In this study, we combine lexical and acoustic markers extracted from a semi-structured conversational setting. To do this, we introduce a stratification method to control for variations in word usage so that acoustic markers can be compared despite vastly different sentence structures and conversational lengths between participants. We hypothesize that the combination of both features improves the ability to distinguish MCI from those with normal cognition. We also hypothesize that semantic features can greatly benefit classification in the semi-structured setting.

## Materials and Methods

### Clinical Trial Data

We obtained transcripts and audio recordings from an ongoing single blind randomized controlled clinical trial (Clinicaltrials.gov: NCT02871921) (14). Briefly, this clinical trial [aka, Internet-Based Conversational Engagement Clinical Trial: www.i-conect.org (I-CONECT)] aims to enhance cognitive functions and psychological well-being of older adults aged 75 and older by means of social interactions using video chats. The trial is funded by the National Institute of Health and developed based on the cumulative epidemiological findings that social isolation is a risk factor of dementia and therefore increasing social interactions may provide a buffer against cognitive decline among older adults (15, 16). The detail protocol and inclusion and exclusion criteria are found elsewhere (14). The ongoing study is an extension of a previous pilot project, conducted in the United States of America, which found promising results in terms of improvement in cognitive functions post-intervention (17). In the ongoing trial, the experimental group receives almost daily (up to 4 times per week) semi-structured conversations (30-min video chats in English) using an internet/webcam for up to 1 year.

In total, there are 160 non-demented older adults [half with normal cognition (NC) and half with mild cognitive impairment (MCI)], that will be recruited. Clinical diagnoses are based on consensus review by neurologists and neuropsychologists using current published diagnostic research criteria (18). Each conversation has a predefined theme (defined based on the image cue shown to the participant), but the participant responses (e.g., word choice, topics of interest, etc.) are allowed to vary in order to provide natural conversational settings. Study recruitment started in July 2018. For the current analysis we used audio and transcribed data from the first 39 participants enrolled in the experimental group available as of March 2020. We used the third day of the first week of video chats for each participant, extracted by an analyst who is independent from the trial project to retain blind status of the participants' randomization to the project statistician and assessors.

#### Consent and Ethical Approval

The study procedures were reviewed and approved by the Institutional Review Board (IRB) at Oregon Health & Science University (OHSU) IRB STUDY00015937 (14). All participants signed the informed consent form.

#### Conversational Details

There were two types of conversations conducted: weekly telephone check-ups (WC) and video chat (VC). WCs involve a short questionnaire that is asked for each participant to monitor their health status including incidence of falls, injury and hospitalization as well as amount of social interactions, and is provided to both control and experimental groups. Most of the questions in WC are responded by yes/no or multiple choice, but some questions are open-ended (e.g., if the participant experienced injury, we ask to describe the nature of the injury or accident). The check-in conversation lasts for roughly 10–15 min. On the other hand, VCs are provided only to those in the experimental group. VCs involve face-to-face conversations conducted remotely by webcam and are semi-structured conversations. Each day of the week a *conversational theme* is offered along with a photograph related to the theme. The participant is asked to choose among *subtopics* that go along with the theme and encouraged to explore topics of their interests, inspired by the photograph. We use both VC and WC recordings for the audio analysis in this study. For linguistic analysis, we utilize a HIPAA-compliant manual transcription service (https://www.transcribeme.com/) to align the audio and provide transcriptions for each conversation.

For the rest of the manuscript, we will refer to WC conversations as “**structured questions with open-ended answers**” to distinguish from traditional “structured conversations” which control for the structure of responses in addition to interviewer questions. We will refer to VC conversations as “**semi-structured conversations**.”

### Feature Representations of Two Modalities (Linguistic Marker Extraction LIWC, Acoustic Marker Extraction MFCC)

#### Linguistic Marker Extraction

Linguistic features are obtained using the 2007 English version of Linguistic Inquiry and Word Count (LIWC) (19), which consists of 4,487 word roots, each of which are labeled with 64 “LIWC categories” [see Table 1 in Pennebaker et al. (19)]. For each conversation, we obtain the 64-dimensional LIWC feature vector for each word in the conversation where each dimension corresponds to a LIWC category (1 = if the word is a member of a category, 0 = otherwise). We then sum over all words in the conversation to obtain a single 64-dimensional feature vector as the *linguistic marker* for the conversation. For illustrative examples of LIWC feature vectors, we refer the reader to Figure 1 of Asgari et al. (1).

#### Acoustic Marker Extraction

Raw acoustic features are 1-dimensional pressure signals sensed by the microphone during the VC recordings. We converted the 1D signals into mel-frequency cepstral coefficients (MFCC) (20) using the librosa library (20, 21). We used a sampling rate of 22,050 Hz, with a windowing procedure of 23ms frames with 10ms step size. This combination results in around 506 samples per second. We used Hamming smoothing between frames. There were 39 features associated with each sample: for each time-series frame, there are 13 MFCC feature bands (δ_0_). We take the first- (δ_1_) and second-order (δ_2_) changes in each band to arrive at a set of 39 total features for each time frame. We then take the minimum (min), maximum (max), mean (avg) and standard deviations (std) of the 39 features for each conversational turn to obtain 156 summary features per turn. Here, we define “turn” as a single round of interviewer and participant responses.

Finally, we take the maximum (MAX), mean (AVG), and standard deviation (STD) *across turns* to arrive at a set of 468 features. Since different conversations have different lengths, we use the following procedure to obtain a fixed 468 dimensional *acoustic marker* for each conversation:

1. For each participant turn in conversation, collect the element-wise *minimum, maximum, mean*, and *standard deviation* for each of the 39 MFCC features across the time samples (39 × 4 = 156 features per turn).
2. Calculate the element-wise *maximum, mean, standard deviation* across all turns in the conversation (156 × 3 =468 features per conversation).
3. For example, suppose a participant speaks for 10 turns. For turn 1, the participant talks for 15 s. With our downsampling procedure, we get 7,590 (506 samples/s × 15 s) observations for turn 1, each consisting of 39 MFCC examples. The max, min, mean and std operators are applied across the 7,590 observations to obtain 156 features (4 × 39) summarizing the audio information contained in turn 1. Similarly, 156 features are obtained for all 10 turns, and max, mean, std operators are calculated to derive a total of 468 (156 × 3) features summarizing the entire conversation.

We note that there are turns in which the speakers (interviewer and participant) overlap. In such cases, we took the beginning of the turn to be the nearest second in which the participant begins speaking.

### MCI Prediction as Binary Classification

We formulate MCI prediction as a binary classification problem (0 = NC, 1 = MCI) for which logistic regression provides an interpretable solution with respect to the linguistic and acoustic features of interest. To prevent overfitting and to encourage sparsity, we use elastic net regularized logistic regression (22) as a baseline model for all classification settings. Taking β to be the variable coefficients, the binary classification objective of our modeling task is formulated as follows:


(1) $$\begin{array}{r} \underset{\beta }{\text{minimize}}\frac{1}{N}\overset{\text{N}}{\underset{i\;=\;1}{\sum }}\overset{d}{\underset{j\;=\;1}{\sum }}L\left(\sigma \left(x_{i}^{\left(j\right)}\cdot \beta_{j}\right),\;y_{i}\right)\;+\;\alpha_{1}||\beta |{|}_{2}^{2}\\ \;+\alpha_{2}\;||\beta |{|}_{1} \end{array}$$

Where σ is the sigmoid function:


$$\begin{array}{r} \sigma \left(z\right)\;=\;\frac{1}{1\;+\;e^{z}} \end{array}$$

and


$$\begin{array}{r} L\left(z,\;y\right)\;=\;-y\log z\;-\;\left(1\;-\;y\right)\log\left(1\;-\;z\right) \end{array}$$

is the logistic loss.

We take α_1_ and α_2_ to be the hyperparameters of the elastic net regularizer, with the *l1-ratio* $\frac{\alpha_{1}}{\alpha_{1}+\alpha_{2}}$ controlling the tradeoff between *l*1 and *l*2 regularization. The rationale behind using the elastic net is that we have a large number of summary acoustic features, many of which may have correlations. Sparsity in the coefficients allows for implicit feature selection among these variables and lends itself to improved interpretability. However, since the *l*1 solution is not unique, the added l2 regularization improves the stability of features selected from different runs of the algorithm. Implementation of the elastic net algorithm is done using the Scikit-Learn library (23).

#### Ensemble Model

We define ensemble features as follows: 1) first, separate elastic net classifiers are trained for each modality, one using LIWC only and one using MFCC only; 2) next, combination features are compiled, combining both LIWC and MFCC features into a 532-dimensional vector; 3) the logits (un-normalized outputs) of the LIWC and MFCC classifiers are concatenated with the combo features to produce a set of *meta-features*; 4) a meta-classifier is trained using the meta-features.

### Interpretation of Coefficients

We define *overlap* as follows: let *B* = (β_1_, …, β_100_) denote the sequence of coefficients for a particular feature β over 100 train-test splits. For each β_*i*_, define the closed interval *I*_*i*_ = [0, β_*i*_] if β_*i*_≥0 and *I*_*i*_ = [β_*i*_, 0] if β_*i*_ < 0. Let = (*I*_1_, …, *I*_100_) denote the sequence of closed intervals corresponding to the coefficient over the 100 train-test runs. Define


$$\begin{array}{r} I^{*}\;=\;\overset{100}{\underset{i\;=\;1}{⋂}}I_{i} \end{array}$$

as the *overlap* between the coefficient interval sequence *I*.

We define feature contributions as follows:


(2) $$\begin{array}{r} \text{Importance}\left(\beta_{i}\right)\;=\;\frac{\left|\beta_{i}\right|}{\sum_{j}|\bar{\beta_{j}}|}, \end{array}$$

where $\bar{\left|\beta_{j}\;\right|}$ is the average magnitude of feature coefficient *j*.

### Subtopic Stratification

Train-test distribution mismatch can lead to poor classification performance. In a typical classification problem, train-test mismatch is often a result of class imbalance. For our problem, however, we have rather balanced MCI/normal cognition (NC) sample ratios. Instead, we have vastly different conversational topics that are covered by conversations. A classifier exposed to different topics during training than in testing can exhibit poor generalization due to train-test mismatch rather than algorithmic reasons. Furthermore, we find that acoustic features obtained on different conversational topics are vastly different, even for the same participant. This is consistent with the idea that there is a tradeoff between variation in language and the fidelity of acoustic markers obtained during automatic extraction (4). Thus, we aim to stratify train-test splits such that both MCI and NC conversations have similar topic distributions during training and testing.

**Figure 1:**
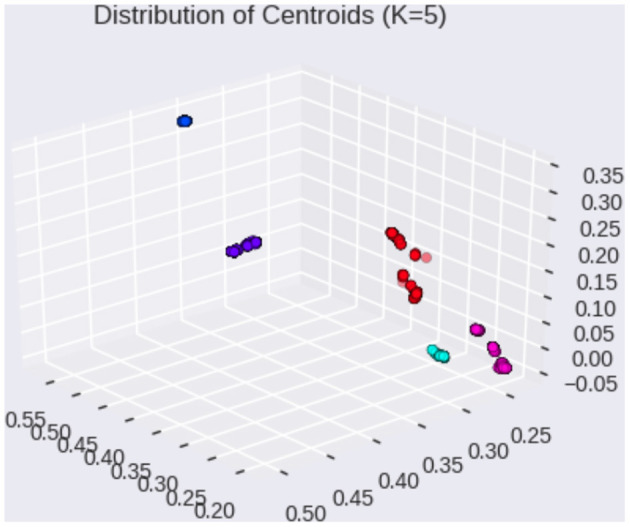
Distribution of cluster centroids on LIWC question vectors (1,000 EM runs).

For example, Figure 1 visualizes the k-means cluster centroids of Linguistic Inquiry and Word Count (LIWC) (19) question vectors obtained from 1,000 runs of the expectation-maximization (EM) algorithm. Since the EM algorithm produces nonunique solutions, repeated runs of the algorithm allow for visualization of the distribution of cluster means. Here, we see that the cluster centroids are distributed tightly across different EM runs, suggesting heterogeneity in the questions (i.e., topics and directions) brought on by the interviewer during various conversations.

In order to resolve this heterogeneity, we propose the following algorithm to stratify participants based on themes and *subtopics* of the conversation. For each participant, there is a central theme of the conversation which is introduced by the interviewer (see Method section for clinical trial description). For example, on the third day of the first week of interventions (see Clinical Trial Data section for detail), the conversational theme is set to “summertime,” and the participants are encouraged to discuss topics related to the summer. By contrast, the second day of the week involves a different theme (sports), leading to a vastly different set of topics of discussion among participants. We thus propose to build a different classifier for different themes. In this study, we constrain the theme of the discussion to “summertime”—a theme that is used for all participants.

We then stratify our train-test split by balanced sampling from these *subtopic clusters* (Figure 1) to minimize imbalance among the conversational topics between train-test distributions. We consider this stratification at the level of “subtopic” since we already account for the theme differences by considering the “summertime” theme. Our subtopic-stratified shuffle split is performed as follows: 1) for K splits, obtain train-test splits using subtopic stratification; 2) for each split, perform hyperparameter tuning using cross validation (CV) on the training set; 3) after hyperparameter tuning, train on the entire training set for the current split using CV hyperparameters; 4) after training, evaluate on test set, record area under the receiver operating curve (AUC Score) and F1 scores for the current split. In total, we consider *K* = 100 stratified shuffle splits using a 80% training and 20% testing ratio, as commonly done in predictive modeling studies (24). We compare the AUC scores by each feature modality (linguistic only, acoustic only, combined features, and ensemble). Since linguistic markers have been shown to be effective in differentiating cognitive status in semi-structured conversations, we use them as baselines for comparison.

## Results

### Participants Characteristics

Demographic characteristics of the participants are summarized in Table 1 below.

**Table 1:** Demographic characteristics by baseline cognitive status.

**Variable**	**MCI group (*n* = 15)**	**NC group (*n* = 17)**	***p*-value**
Age	79.3 (3.7)	80.0 (4.3)	0.72
Gender (% Women)	53.3%	82.4%	0.08
Years of Education	14.8 (2.8)	15.8 (3.0)	0.36
Race (% White)	86.7%	94.1%	0.49
MoCA Score (25)	21.3 (2.9)	25.7 (2.5)	0.00018

*MCI, mild cognitive impairment; NC, those with normal cognition. Two-sample student t-tests for continuous variables and Pearson chi-squared test for categorical variables were used to calculate p-values*.

### Model Comparison

Using the subtopic stratification scheme, we compare the performance of several modalities in Table 2. The linguistic feature setting refers to the elastic net classifier using only Linguistic Inquiry and Word Count (LIWC) features for both training and testing. The acoustic feature setting uses only mel-frequency cepstral coefficients (MFCC) features (see Feature Representation of Two Modalities in Materials and Methods). Combo features refer to the simple concatenation of LIWC and MFCC features, resulting in a 532-dimensional representation of each participant conversation (64 dimensions for linguistic+ 468 dimensions for acoustic). For each feature representation, elementwise standardization is fitted to the training set and applied as a preprocessing step before classification.

**Table 2:** Comparisons of behavioral marker performances on 100 subtopic-stratified shuffle splits using semi-structured conversations.

**Feature**	**AUC Score**	**t-statistic**	***p*-value**
Linguistic	74.9 (3.27)	-	-
Acoustic	64.9 (4.66)	−3.52	5.37e-4
Combo	79.9 (4.37)	1.78	0.077
Ensemble	**82.7 (3.53)**	**2.97**	**0.003**

*Features are obtained from video chats (semi-structured conversations). Variance across different splits is reported in parenthesis. Bolded: the best performing model by AUC Score*.

Only the performances on the test sets are reported since training performance does not measure generalization capacity. The mean test AUC scores are reported, with the variance across different train-test splits reported in parenthesis in Table 2. We report the two-sample *t*-test results, comparing various feature modalities against the linguistic-only baseline. In total, we consider K=100 different stratified train-test splits.

In Table 2, we consider linguistic marker performance to be the baseline for comparison. Using acoustic markers alone, we see a notable decrease in performance. This is because unlike previous papers such as Roark et al. (4) and Alhanai et al. (13), the semi-structured nature of our conversations introduces many sources of noise not encountered in structured conversational settings. For example, VC (video chat) conversations last anywhere from 9 to 25 min in terms of total participant speaking time. This means that the same sampling and statistical averaging techniques may in fact result in different levels of feature granularity across patients. Additionally, the phonetic information provided by conversations largely depends on the spoken words used to generate them; semi-structured conversations result in a much larger variance in terms of word coverage as well as the pace of conversation that is not observed in structured conversations. The result of these complications can be observed in acoustic marker performance: using MFCCs alone results in larger variance across splits and lower overall prediction power.

However, combining the linguistic and acoustic markers result in a notable performance boost compared to using either linguistic or acoustic markers alone. Although combo features provided statistically significant improvement compared to acoustic features, they outperform linguistic features only marginally. For this reason, we introduce the ensemble setting to reduce the variance of our predictions, which in turn produces over 80% AUC with statistically significant (*p* < 0.01) improvement compared to the linguistic marker baseline.

### LIWC Markers: Stability of Linguistic Feature Selections

**Figure 2:**
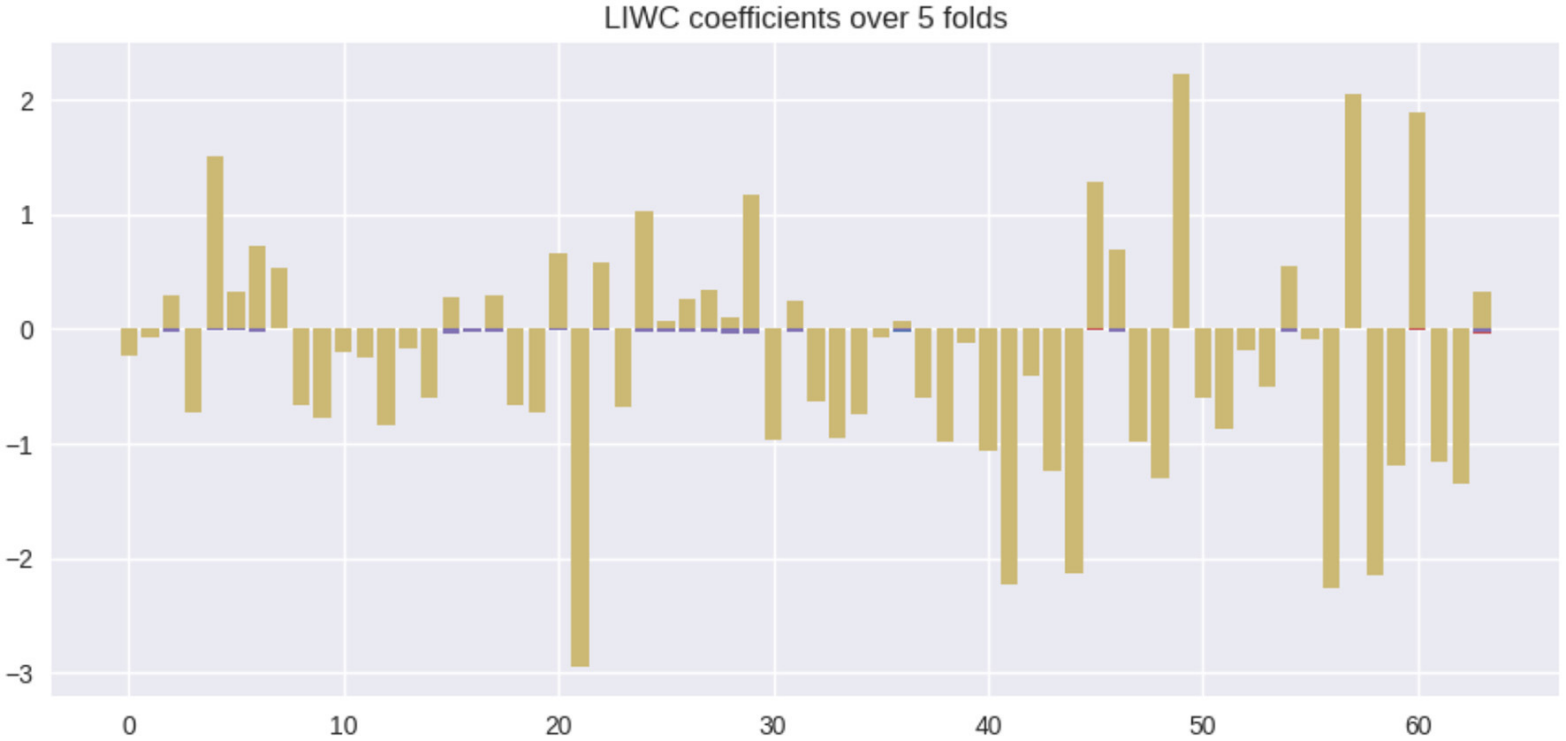
Feature coefficients β across various train-test splits. Colors: yellow = overlapping feature weights, purple = non overlapping feature weights.

In this section, we quantify the feature contributions of various linguistic markers for MCI prediction and provide an interpretation of their potential associations with the diagnosis. Figure 2 summarizes the feature coefficient changes across the train-test splits. Coefficients for each individual split are plotted as purple bars. For each feature, the coefficient overlap (defined in methods section) is visualized in yellow. From Figure 2, we see that the majority of the coefficient weights overlap across various train-test splits, despite degrees of train-test distribution mismatch between splits. This observation indicates the stability of linguistic features selected by L1.

In total, there are 22 features that positively correlate with MCI and 42 features that negatively correlate with MCI (NC correlated). We list the top 10 ranking features (in terms of coefficient weights) of each set in Table 3 below. The listed features correspond to LIWC categories whose semantic meanings are outlined in Table 1 of Pennebaker et al. (19). Among the MCI correlated features, the LIWC category “death”—i.e., words related to one's *personal concerns* of death—possesses the highest odds ratio (67% increase in odd per unit of increase). In parenthesis, we report the 95% confidence interval (CI) for the odds ratio column. Here, each “unit” of increase for any LIWC feature is one *standard deviation* change in the LIWC categorical count. For example, adding a single word “kill” to a person's dialog responses will not change the person's “death” LIWC category feature, but adding multiple responses involving one's preoccupation with death (e.g., deterioration, funeral) can potentially lead to a unit increase if words belonging to the “death” LIWC category is overrepresented by an additional standard deviation compared to the rest of the conversations in the training set. As a baseline, an odds ratio of 1.0 neither increases nor decreases the odds of an MCI prediction.

**Table 3:** Top 10 LIWC feature coefficients correlated with MCI compared with top 10 coefficients correlated with NC.

**Features associated with MCI**	**Coeff**.	**Odds Ratio**	**Features associated with NC**	**Coeff**.	**Odds ratio**
Death	0.50	1.67 (1.6–1.7)	Swear	−0.74	0.48 (0.4–0.5)
They	0.49	1.67 (1.6–1.7)	Feel	−0.61	0.55 (0.5–0.6)
Home	0.38	1.48 (1.4–1.5)	Percept	−0.56	0.58 (0.5–0.6)
Ingest	0.38	1.47 (1.4–1.5)	Nonfl	−0.54	0.59 (0.5–0.6)
Number	0.32	1.40 (1.4–1.4)	Insight	−0.53	0.59 (0.5–0.6)
Friend	0.30	1.37 (1.3–1.4)	Leisure	−0.49	0.62 (0.6–0.6)
You	0.24	1.28 (1.2–1.3)	Assent	−0.46	0.64 (0.6–0.6)
Social	0.24	1.27 (1.2–1.3)	Anger	−0.44	0.65 (0.6–0.7)
We	0.21	1.25 (1.2–1.3)	Money	−0.39	0.68 (0.6–0.8)
Bio	0.20	1.22 (1.2–1.2)	Time	−0.31	0.73 (0.7–0.8)

*Ninety five percent confidence interval is given for odds ratio*.

Interestingly, we find that MCI and NC patients differ greatly in the “personal concerns” meta-category, consisting of *work, leisure, home, money, relig[ion]*, and *death* [more details in Table 1 of Pennebaker et al. (19)]. Specifically, the categories *leisure* and *money* decrease the odds of being MCI by 30%+ whereas *death* and *home* increases the odds by 40%+. We also find that unit increases in categories related to “social processes” (another meta-category) such as *ingest* (food, drinks), *friend, we*, and *social* increase the odds of being MCI by at least 27%. On the other hand, unit increases in “informal language” use (another meta-category) such as *swear* (words), *nonfl[uent]* (e.g., laughter) decrease the odds by 40%+. A full list of features, their coefficients and odd ratios are listed in Supplementary Table 1.

### MFCC Markers

**Figure 3:**
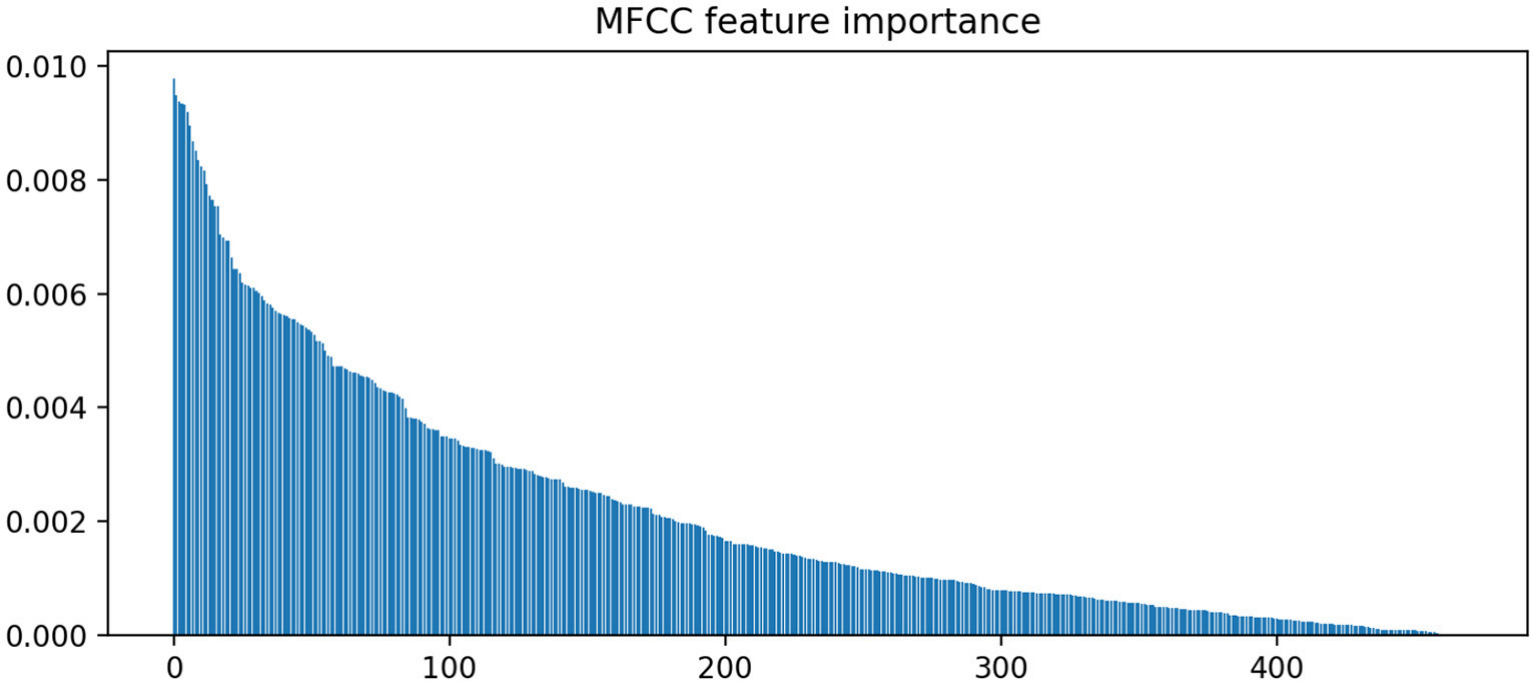
Feature importance rankings for MFCC coefficient weights. Elastic net was used to stabilize the L1 path across different train-test splits.

In contrast to LIWC markers, acoustic markers show more fluctuation in coefficient weights across various splits. Figure 3 illustrates the importance of various MFCC weight coefficients used by the classifier to predict MCI. It is notable that the sparsity pattern is quite different; the large number of features leads to an overdetermined system with correlated features. As a result, L1 regularization selects for only a small subset of contributory features (i.e., coefficients with non-zero weights). Because L1 paths are non-unique, the use of elastic-net regularization stabilizes the sparsity patterns across different train-test splits, as evidenced by the tighter confidence intervals around the odds ratio estimates for each feature. In total, there are 400 non-zero coefficients (averaged across the splits) that significantly contributed to MCI predictions (1.0 not included in confidence interval). However, Figure 3 shows that the weight of their contributions drop off exponentially, with the top 100 features accounting for most of the contributions.

In Figure 3, we find that the feature importances (defined in Methods section) drop below 0.5% after the top 100 ranking features. Thus, we report the top 5 features in terms of feature importance in Table 3.

The MFCC feature coefficients can be interpreted as follows: the MFCC bands increase in frequency e.g., δ_0_-3 represents a lower frequency than δ_0_-4. The first 2 bands, δ_0_-1 and δ_0_-2 represent the total energies of the speech sample (i.e., do not correspond to specific frequencies). Since δ_1_ features represent *instantaneous changes* in the original feature, δ_1_-*x* would correspond to the change in frequency *x* during the speech sample. When averaged across speech samples, avg-δ_0_-*x*, avg-δ_1_-*x* and avg-??_2_-*x* represent the average *amplitude, change in amplitude, and rate of change in amplitude of the frequency x*, respectively, for a spoken turn consisting of a set of speech samples. For a participant, MAX-avg-δ_0_-*x* would represent the maximum over the value averages of the frequency *x* across all spoken turns in the conversation. As an example, consider the feature MAX-min-δ_1_-6. This feature gives the empirical upper bound (MAX) on the minimum (min) rate of change (δ_1_) in frequency band number 6. On the other hand, AVG-min-δ_1_-6 gives the empirical average of the lowest rate of change in frequency band 6. Using these features, we can give fine-grained interpretation over how the MFCC features change across turns across different participants over the course of conversations.

In Table 4, we find that the average minimum and maximum amplitudes of frequency band 8 has the highest statistically significant odds ratio. This can be interpretated as follows: for each unit increase (again, in terms of standard deviation) in the minimum or maximum amplitude of frequency band 8 leads to a 16% increase in the odds of a positive MCI prediction. On the other hand, we find that the *lower bound* (MAX-min) on the change (δ_1_) and rates of change (δ_2_) in multiple frequency bands significantly decrease the odds of a positive MCI prediction. The statistical significance of lower bounds (across turns) of frequency band changes may be interpreted as follows: the *min* part of MAX-min corresponds to the minimum of speech changes *within turns*. Maximizing across turns gives a lower bound (a greatest lower bound) on how much a frequency band changes over the entire conversation. For example, a unit increase in MAX-min-δ_1_-13 from 0.0 to 1.0 would mean that the frequency band 13 fluctuates at a rate at least 1 standard deviation above the training set mean in any 1 of the participant's responses. The fact that MAX-min-δ_1_-13 has an odds ratio of 0.86 suggests that a unit increase in the lower bound decreases the odds of a positive MCI prediction by 14%. Since frequency band 13 has the highest frequency on the MFCC spectrum, this finding suggests that an increase in the variability (δ_1_) of the highest speech frequency lowers the odds of MCI prediction. Supplementary Table 2 gives the full MFCC features list, coefficient weights and odds ratio (with CI bounds).

**Table 4:** Top five MFCC feature coefficients associated with MCI compared to the top five feature coefficients associated with NC.

**Features associated with MCI**	**Coeff**.	**Odds Ratio**	**Features associated with NC**	**Coeff**.	**Odds Ratio**
AVG, min, δ_0_-8	0.15	1.16 (1.16–1.17)	MAX, min, δ_1_-13	−0.15	0.86 (0.86–0.87)
AVG, max, δ_0_-8	0.14	1.15 (1.15 - 1.15)	MAX, min, δ_0_-7	−0.15	0.86 (0.86–0.87)
AVG, std, δ_0_-1	0.13	1.14 (1.13 - 1.14)	MAX, min, δ_1_-11	−0.15	0.86 (0.86–0.87)
MAX, std, δ_0_-12	0.12	1.13 (1.13 - 1.14)	STD, min, δ_2_-8	−0.15	0.86 (0.86–0.87)
STD, max, δ_0_-2	0.12	1.13 (1.12 - 1.13)	MAX, avg, δ_1_-10	−0.14	0.87 (0.86–0.87)

*Ninety five percent confidence interval is given for the odds ratio*.

### Comparison With Structured Conversations

Finally we compare the performance of features obtained from structured conversations in Table 5. Here, both text transcripts and audio signals are obtained from Weekly Check-in (WC) conversations (i.e., structured conversations). Similar to semi-structured VCs, we use a single conversation from Week 1 for each participant for analysis. The same set of participants are used for WC analysis as those used for VC analysis. A comparison of Tables 2, 5 reveals that the AUC for each feature setting performed significantly decreased in the structured setting compared to the semi-structured setting. We compare with the VC linguistic feature performance as baseline for hypothesis testing.

**Table 5:** Comparisons of behavioral marker performances on 100 subtopic-stratified shuffle splits using structured conversations.

**Feature**	**AUC Score**	**t-statistic**	***p*-value**
Linguistic	56.8 (6.3)	−7.30	7.37e-12
Acoustic	55.8 (6.3)	−6.58	4.2e-10
Combo	49.5 (4.4)	−10.08	1.7e-19
Ensemble	56.0 (7.2)	−6.24	2.64e-9

*Features obtained from Weekly Check-ins (structured conversations). Variance across different splits is reported in parenthesis*.

One possible explanation for this drop in performance is that almost all of the advantages of linguistic features are lost in structured conversation. We can see that including linguistic predictions as input to the ensemble model did not increase the performance at all. In fact, we find that the inclusion of linguistic features into the combo model led to overfitting and consequently drop in performance. It is interesting to note that the classifiers exclusively using acoustic features also decreased in performance. Although the *prompts* used by the interviewers during WCs are fixed, the answers given by the participants may differ (although not nearly as much as VC responses), e.g., “I am doing fine” vs. “I am not well” in response to “how do you feel this morning?”

## Discussion

In this study, we examine the use of linguistic and acoustic features for MCI classification in semi-structured conversations. The topics of discussion, duration and pace of speech in structured conversations do not vary as much as they do in unstructured conversations since structured conversation are responses to the same set of questions. As a result, semi-structured conversations introduce challenges in the stability of behavioral markers, especially for ones dependent on acoustic signals. By combining both acoustic and linguistic markers, we show that the composite behavioral markers can significantly out-perform any single modality alone. Using the elastic net, we show that the feature coefficients reveal some interesting differences in lexical preference and speech patterns between MCI and NC groups.

In semi-structured conversations, we see the benefit of using linguistic features that capture semantic meanings of responses (LIWC). In VC conversations, the LIWC, which addresses linguistic features, significantly outperform the MFCC, which addresses acoustic features (AUC 0.75 vs. 0.65). By contrast, when structured questions are used in the WC setting, linguistic features lose their advantages, and we do not see a synergistic effect between the two modalities in either the combo or the ensemble models. The key difference between the VC and WC setting is that the participants were allowed to guide the conversation instead of the interviewer in the VC. That is, the differences in the linguistic markers were a result of the degree of freedom in the conversation content and topic selection. This finding seems to support our hypothesis that semantic features can greatly benefit classification in the semi-structured setting.

Compared with other research groups which analyzed structured conversations using acoustic features, such as Roark et al. (which found AUC between 0.63 and 0.73), and Frasier et al. (AUC 0.88 for combined modalities), our performance in structured conversation using linguistic features in differentiating MCI from those with normal cognition was lower (AUC of 0.56–0.57). This suggests that linguistic features that rely heavily on semantic meanings do not perform well for analyzing structured conversations. Additionally, Frazier et al. also included eye tracking features which could have contributed to the higher AUC in detecting MCI.

Additionally, in previous structured studies, the acoustic features that perform well are extracted from different speakers saying the exact same sentences or describing the same visual prompts. WC conversations in our study used open-ended questions, so the answers may still vary between participants, although not nearly as much as in semi-structured conversations where they drive the conversation. For example, one question is “did you visit the hospital last week”—a yes or no question, but the follow up question asks the reason of the visit which may lead to very different responses. That is, our structured conversations may not be as structured as exact sentences spoken or fixed visual inputs. Thus, while there is a performance drop to acoustic-only classifiers (0.65–0.55), the difference is not as much as the performance drop in the linguistic (0.75–0.56) and ensemble (0.83–0.57) ones.

In this study, we used two unique approaches to improve the ability to detect MCI when semi-structured conversations are analyzed. First, although semi-structured conversations had more variations in conversational content, we were able to compare across a diverse set of conversational structures using sub-topic stratification to minimize the distribution mismatch between train-test splits. An example of train-test mismatch is when the conversational topics in the training set are drastically different from the test set. For example, if no training set conversations involved any discussion of leisure activities, then it is likely that the *leisure* LIWC category would not contribute as much to the classification decision. In other words, sub-topic stratification ensures that the distribution of over the feature space (approximated by the distribution over LIWC features) is similar for training and test set samples.

Second, we improved the predictive performance of combined acoustic and linguistic features through the use of ensemble. While we showed in Table 2 that combined features alone can significantly improve the classification performance compared to either linguistic and acoustic features alone, ensemble can even further improve the performance by directly combining the linguistic and acoustic features (combo model). This is largely because of the effect of ensemble on variance; by combining the outputs of multiple models, the variance can decrease when compared to the individual variances of each model in the ensemble (22, 26). Interestingly, model ensemble does not only apply to generalized linear models such as logistic regression but also to decision tree methods, which can be closer to the typical representation in clinical decision algorithms. In this study, we chose logistic regression as the classifier for each of the modalities because its feature coefficients permit useful odds-ratio interpretations of the features involved. In future work, it may be interesting to combine logistic regression outputs of the different modalities with tree-based classifiers (e.g., random forests) that uses these classifier decisions as meta-features.

As for the differences in lexical preference and speech patterns between MCI and NC groups, we revealed an interesting finding. For example, an analysis of the odds ratios of LIWC features suggests that the LIWC meta-category “personal concerns” distinguish between topics that are preferred by MCI participants (topics related to health concerns related to home, deterioration and death) from those preferred by NC (topics related to leisure and money). The fact that different LIWC categories have different predictive value suggests that *interviewer questions*, prioritizing different topics of discussion, can potentially drive conversations with vastly different prediction outcomes. For example, interview questions that lead participants to divulge their personal concerns can potentially be more informative LIWC feature vectors compared to interview questions that focus on occupation (*work* category, OR = 1.09) or past achievements (*achieve* category, OR = 0.99).

One limitation of our approach is that the degree of train-test distribution mismatch is highly dependent on the range of conversational topics covered in the set of conversations. For this study, we constrained the theme to be “summertime” and dealt with subtopic imbalance by stratified sampling. However, in a real-world setting, unstructured conversations can span much larger sets of topics and dialog structures. By conditioning on select topics and by instilling certain interview structures, we likely cannot generalize to the unstructured setting. However, we illustrate that interpretable insights can be obtained by imposing some dialog structure but allowing the participants to explore the linguistic space without strong constraints.

Another limitation of this study is the use of manual transcriptions for speech-to-text translation. We used manually transcribed conversational data, which is time-consuming and costly. However, our refined ASR is already available (27), and we plan to apply the ASR to our entire speech sample and replicate the current analyses as a validation study.

Future studies can build upon our findings by comparing differences in language behavioral markers under other dialog structures and themes. It is possible that we may not need to construct a single classifier that generalizes to the unstructured setting since the space of possible topics and dialog structures is simply intractable to estimate. Instead, we show that it may be possible to build many classifiers under various semi-structured settings that can be combined synergistically under different conversational settings.

One of the potential benefits of accurate diagnostic predictions in the semi-structured settings is its practical use in the greater community (outside of clinical settings). For example, MCI diagnosis is currently done at outpatient clinics, using extensive neuropsychology testing. Early diagnostic screening using semi-structured conversations can potentially be carried out even before scheduled outpatient visits or used to monitor daily changes longitudinally.

This study is among the first to examine semi-structured conversations (as opposed to structured conversations) and to extract both acoustic and linguistic features associated with MCI diagnosis. The results are still preliminary because the sample size is small, and the generalizability is limited. However, in the future, our approach may directly assist in development of a useful app where conversations can be analyzed longitudinally and identify those at high risk of cognitive decline associated with dementia. This type of identification approach of early-stage dementia which does not require clinical visits or expensive biomarkers analyses can be of significant public health importance.

## Data Availability Statement

The datasets presented in this study can be found in online repositories. The names of the repository/repositories and accession number(s) can be found below: https://figshare.com/projects/Frontiers_Submission/118467. The examples of audio and video data are found at https://www.i-conect.org/request-data.

## Author Contributions

FT wrote the code, conducted experiments, and was the primary preparer of the manuscript. JC contributed to discussions, performed experiments on related datasets to check methodology, and analysis methods. HD is the principal investigator of the clinical trial, provided the data, research hypotheses, feedback, and edits for this manuscript. JZ is the main contributor for experiment design and direction of study, providing feedback, and edits for this manuscript. All authors contributed to the article and approved the submitted version.

## Funding

This study was supported by the NIH under grants: RF1AG072449, R01AG051628, R01AG056102, R01AG033581, P30AG066518, the National Science Foundation under Grant IIS-1749940, Office of Naval Research N00014-20-1-2382.

## Conflict of Interest

The authors declare that the research was conducted in the absence of any commercial or financial relationships that could be construed as a potential conflict of interest.

## Publisher's Note

All claims expressed in this article are solely those of the authors and do not necessarily represent those of their affiliated organizations, or those of the publisher, the editors and the reviewers. Any product that may be evaluated in this article, or claim that may be made by its manufacturer, is not guaranteed or endorsed by the publisher.
