# Supplementary material for: The Joint Effects of Acoustic and Linguistic Markers for Early Identification of Mild Cognitive Impairment
Source: Front Digit Health. 2022 Feb 11;3:702772. doi: 10.3389/fdgth.2021.702772 (PMC8878676; doi:10.3389/fdgth.2021.702772)
Supplement: Supplementary file 1 [file Data_Sheet_1.docx]

### Supplemental Materials

**Supplemental Table 1.** Comprehensive LIWC feature coefficients analysis. Coefficients were averaged over models derived from 100 randomized train-test splits. Each feature corresponds to one of the 64 LIWC categories, see Table 1. LIWC2015 Output Variable Information in Pennebaker et al. (*13*) for examples.

| Rank | Feature | Mean Coefficient | % Contribution (MCI) | Odds Ratio | CI |
| --- | --- | --- | --- | --- | --- |
| 1 | death | 0.50 | 7.1 | 1.67 | 1.62 - 1.71 |
| 2 | they | 0.49 | 6.9 | 1.67 | 1.59 - 1.74 |
| 3 | home | 0.38 | 5.4 | 1.48 | 1.44 - 1.52 |
| 4 | ingest | 0.38 | 5.3 | 1.47 | 1.44 - 1.50 |
| 5 | number | 0.32 | 4.5 | 1.40 | 1.35 - 1.44 |
| 6 | friend | 0.30 | 4.3 | 1.37 | 1.33 - 1.40 |
| 7 | you | 0.24 | 3.4 | 1.28 | 1.26 - 1.29 |
| 8 | social | 0.24 | 3.4 | 1.27 | 1.26 - 1.29 |
| 9 | we | 0.21 | 3.0 | 1.25 | 1.22 - 1.28 |
| 10 | bio | 0.20 | 2.8 | 1.22 | 1.21 - 1.24 |
| 11 | hear | 0.11 | 1.6 | 1.13 | 1.10 - 1.16 |
| 12 | work | 0.08 | 1.2 | 1.09 | 1.07 - 1.12 |
| 13 | shehe | 0.06 | 0.8 | 1.06 | 1.04 - 1.08 |
| 14 | ppron | 0.05 | 0.7 | 1.05 | 1.04 - 1.06 |
| 15 | body | 0.03 | 0.4 | 1.04 | 1.01 - 1.06 |
| 16 | anx | 0.03 | 0.4 | 1.04 | 1.01 - 1.07 |
| 17 | posemo | 0.01 | 0.1 | 1.01 | 0.99 - 1.03 |
| 18 | sad | 0.00 | 0.1 | 1.01 | 0.99 - 1.03 |
| 19 | achieve | -0.01 | -0.1 | 0.99 | 0.98 - 1.01 |
| 20 | pronoun | -0.02 | -0.3 | 0.98 | 0.97 - 0.99 |
| 21 | preps | -0.03 | -0.5 | 0.97 | 0.96 - 0.98 |
| 22 | incl | -0.04 | -0.5 | 0.97 | 0.95 - 0.98 |
| 23 | affect | -0.05 | -0.7 | 0.95 | 0.94 - 0.97 |
| 24 | certain | -0.06 | -0.8 | 0.95 | 0.93 - 0.97 |
| 25 | conj | -0.06 | -0.9 | 0.94 | 0.93 - 0.95 |
| 26 | tentat | -0.07 | -1.0 | 0.93 | 0.92 - 0.95 |
| 27 | humans | -0.08 | -1.2 | 0.92 | 0.91 - 0.94 |
| 28 | space | -0.08 | -1.2 | 0.92 | 0.91 - 0.93 |
| 29 | present | -0.09 | -1.2 | 0.92 | 0.91 - 0.93 |
| 30 | auxverb | -0.09 | -1.2 | 0.92 | 0.91 - 0.92 |
| 31 | sexual | -0.09 | -1.3 | 0.92 | 0.89 - 0.95 |
| 32 | funct | -0.11 | -1.5 | 0.90 | 0.90 - 0.90 |
| 33 | adverb | -0.13 | -1.8 | 0.88 | 0.87 - 0.90 |
| 34 | health | -0.13 | -1.8 | 0.88 | 0.87 - 0.90 |
| 35 | future | -0.13 | -1.8 | 0.88 | 0.86 - 0.91 |
| 36 | verb | -0.13 | -1.8 | 0.88 | 0.87 - 0.88 |
| 37 | ipron | -0.13 | -1.9 | 0.88 | 0.87 - 0.89 |
| 38 | inhib | -0.17 | -2.3 | 0.85 | 0.83 - 0.87 |
| 39 | relativ | -0.18 | -2.6 | 0.83 | 0.82 - 0.84 |
| 40 | negate | -0.20 | -2.8 | 0.83 | 0.80 - 0.85 |
| 41 | motion | -0.20 | -2.9 | 0.82 | 0.81 - 0.83 |
| 42 | family | -0.22 | -3.1 | 0.81 | 0.78 - 0.83 |
| 43 | article | -0.22 | -3.1 | 0.81 | 0.79 - 0.82 |
| 44 | cogmech | -0.23 | -3.2 | 0.80 | 0.79 - 0.81 |
| 45 | see | -0.23 | -3.2 | 0.80 | 0.78 - 0.81 |
| 46 | negemo | -0.24 | -3.4 | 0.79 | 0.77 - 0.80 |
| 47 | excl | -0.25 | -3.5 | 0.78 | 0.77 - 0.80 |
| 48 | filler | -0.26 | -3.7 | 0.77 | 0.76 - 0.79 |
| 49 | cause | -0.27 | -3.7 | 0.77 | 0.75 - 0.80 |
| 50 | i | -0.28 | -3.9 | 0.76 | 0.75 - 0.78 |
| 51 | past | -0.30 | -4.2 | 0.74 | 0.73 - 0.76 |
| 52 | quant | -0.30 | -4.2 | 0.74 | 0.73 - 0.75 |
| 53 | discrep | -0.30 | -4.3 | 0.74 | 0.73 - 0.75 |
| 54 | relig | -0.31 | -4.4 | 0.75 | 0.71 - 0.78 |
| 55 | time | -0.31 | -4.4 | 0.73 | 0.72 - 0.75 |
| 56 | money | -0.39 | -5.4 | 0.68 | 0.67 - 0.70 |
| 57 | anger | -0.44 | -6.1 | 0.65 | 0.63 - 0.67 |
| 58 | assent | -0.46 | -6.4 | 0.64 | 0.62 - 0.66 |
| 59 | leisure | -0.49 | -6.8 | 0.62 | 0.61 - 0.63 |
| 60 | insight | -0.53 | -7.5 | 0.59 | 0.57 - 0.61 |
| 61 | nonfl | -0.54 | -7.6 | 0.59 | 0.57 - 0.60 |
| 62 | percept | -0.56 | -7.8 | 0.58 | 0.56 - 0.59 |
| 63 | feel | -0.61 | -8.5 | 0.55 | 0.53 - 0.57 |
| 64 | swear | -0.74 | -10.4 | 0.48 | 0.46 - 0.50 |

**Supplemental Table 2.** Comprehensive MFCC feature coefficients analysis. Coefficients were averaged over models derived from 100 randomized train-test splits.

| Rank | Feature | Coefficient | % Contribution | Odds Ratio | CI |
| --- | --- | --- | --- | --- | --- |
| 1 | MEAN-min-𝛿0-8 | 0.15 | 33.7 | 1.16 | 1.16 - 1.17 |
| 2 | MEAN-max-𝛿0-8 | 0.14 | 30.8 | 1.15 | 1.15 - 1.15 |
| 3 | MEAN-std-𝛿0-1 | 0.13 | 28.5 | 1.14 | 1.13 - 1.14 |
| 4 | MAX-std-𝛿0-12 | 0.12 | 27.7 | 1.13 | 1.13 - 1.14 |
| 5 | STD-max-𝛿0-2 | 0.12 | 27.0 | 1.13 | 1.12 - 1.13 |
| 6 | MEAN-min-𝛿1-9 | 0.12 | 26.8 | 1.13 | 1.12 - 1.13 |
| 7 | STD-min-𝛿1-1 | 0.12 | 26.6 | 1.13 | 1.12 - 1.13 |
| 8 | STD-mean-𝛿0-5 | 0.11 | 24.9 | 1.12 | 1.11 - 1.12 |
| 9 | MAX-mean-𝛿0-5 | 0.11 | 24.5 | 1.12 | 1.11 - 1.12 |
| 10 | MEAN-std-𝛿1-1 | 0.11 | 23.5 | 1.11 | 1.11 - 1.11 |
| 11 | STD-mean-𝛿2-1 | 0.10 | 23.0 | 1.11 | 1.10 - 1.11 |
| 12 | STD-min-𝛿0-1 | 0.10 | 22.8 | 1.11 | 1.10 - 1.11 |
| 13 | STD-std-𝛿0-13 | 0.10 | 22.5 | 1.11 | 1.10 - 1.11 |
| 14 | STD-std-𝛿0-12 | 0.10 | 21.5 | 1.10 | 1.10 - 1.11 |
| 15 | MAX-std-𝛿0-1 | 0.09 | 20.9 | 1.10 | 1.10 - 1.10 |
| 16 | MAX-max-𝛿0-9 | 0.09 | 20.4 | 1.10 | 1.09 - 1.10 |
| 17 | STD-max-𝛿1-1 | 0.09 | 20.4 | 1.10 | 1.09 - 1.10 |
| 18 | STD-max-𝛿0-9 | 0.09 | 20.4 | 1.10 | 1.09 - 1.10 |
| 19 | MAX-mean-𝛿1-4 | 0.09 | 20.0 | 1.09 | 1.09 - 1.10 |
| 20 | STD-std-𝛿0-9 | 0.09 | 19.8 | 1.09 | 1.09 - 1.10 |
| 21 | STD-std-𝛿0-4 | 0.09 | 19.5 | 1.09 | 1.09 - 1.10 |
| 22 | STD-std-𝛿0-7 | 0.09 | 19.1 | 1.09 | 1.09 - 1.09 |
| 23 | MAX-std-𝛿0-7 | 0.09 | 19.1 | 1.09 | 1.09 - 1.09 |
| 24 | MAX-max-𝛿2-13 | 0.08 | 18.8 | 1.09 | 1.09 - 1.09 |
| 25 | MAX-max-𝛿0-8 | 0.08 | 18.1 | 1.09 | 1.08 - 1.09 |
| 26 | MEAN-mean-𝛿1-1 | 0.08 | 17.5 | 1.08 | 1.08 - 1.08 |
| 27 | MEAN-max-𝛿0-11 | 0.08 | 17.4 | 1.08 | 1.08 - 1.09 |
| 28 | STD-min-𝛿0-5 | 0.08 | 17.4 | 1.08 | 1.08 - 1.08 |
| 29 | MAX-min-𝛿0-8 | 0.08 | 17.2 | 1.08 | 1.08 - 1.08 |
| 30 | STD-std-𝛿1-7 | 0.08 | 17.1 | 1.08 | 1.08 - 1.08 |
| 31 | STD-std-𝛿1-9 | 0.07 | 16.6 | 1.08 | 1.07 - 1.08 |
| 32 | STD-mean-𝛿0-6 | 0.07 | 16.2 | 1.08 | 1.07 - 1.08 |
| 33 | MAX-max-𝛿0-13 | 0.07 | 15.9 | 1.07 | 1.07 - 1.08 |
| 34 | MAX-min-𝛿2-7 | 0.07 | 15.9 | 1.07 | 1.07 - 1.08 |
| 35 | MEAN-min-𝛿0-11 | 0.07 | 15.2 | 1.07 | 1.07 - 1.07 |
| 36 | MAX-max-𝛿1-4 | 0.07 | 15.1 | 1.07 | 1.06 - 1.08 |
| 37 | MAX-max-𝛿1-9 | 0.07 | 14.8 | 1.07 | 1.06 - 1.07 |
| 38 | MEAN-min-𝛿1-2 | 0.07 | 14.7 | 1.07 | 1.06 - 1.07 |
| 39 | MEAN-std-𝛿2-1 | 0.07 | 14.5 | 1.07 | 1.07 - 1.07 |
| 40 | MAX-mean-𝛿0-8 | 0.07 | 14.5 | 1.07 | 1.06 - 1.07 |
| 41 | MEAN-mean-𝛿2-1 | 0.06 | 14.3 | 1.07 | 1.06 - 1.07 |
| 42 | STD-max-𝛿2-9 | 0.06 | 14.2 | 1.07 | 1.06 - 1.07 |
| 43 | STD-max-𝛿1-9 | 0.06 | 14.1 | 1.07 | 1.06 - 1.07 |
| 44 | STD-std-𝛿2-12 | 0.06 | 13.4 | 1.06 | 1.06 - 1.07 |
| 45 | MEAN-max-𝛿1-10 | 0.06 | 13.4 | 1.06 | 1.06 - 1.07 |
| 46 | MEAN-max-𝛿0-5 | 0.06 | 13.1 | 1.06 | 1.06 - 1.06 |
| 47 | MEAN-max-𝛿1-6 | 0.06 | 13.1 | 1.06 | 1.06 - 1.06 |
| 48 | STD-max-𝛿1-2 | 0.06 | 12.9 | 1.06 | 1.06 - 1.06 |
| 49 | STD-std-𝛿1-1 | 0.06 | 12.9 | 1.06 | 1.06 - 1.06 |
| 50 | MAX-max-𝛿1-7 | 0.06 | 12.8 | 1.06 | 1.06 - 1.06 |
| 51 | MAX-max-𝛿1-8 | 0.06 | 12.8 | 1.06 | 1.05 - 1.06 |
| 52 | STD-std-𝛿1-4 | 0.06 | 12.7 | 1.06 | 1.06 - 1.06 |
| 53 | STD-std-𝛿1-5 | 0.06 | 12.3 | 1.06 | 1.05 - 1.06 |
| 54 | MAX-min-𝛿0-13 | 0.05 | 12.1 | 1.06 | 1.05 - 1.06 |
| 55 | MEAN-min-𝛿0-5 | 0.05 | 11.9 | 1.06 | 1.05 - 1.06 |
| 56 | MEAN-std-𝛿0-9 | 0.05 | 11.8 | 1.05 | 1.05 - 1.06 |
| 57 | STD-min-𝛿2-1 | 0.05 | 11.5 | 1.05 | 1.05 - 1.06 |
| 58 | STD-min-𝛿0-9 | 0.05 | 11.4 | 1.05 | 1.05 - 1.06 |
| 59 | STD-std-𝛿0-1 | 0.05 | 11.4 | 1.05 | 1.05 - 1.06 |
| 60 | STD-max-𝛿2-1 | 0.05 | 11.4 | 1.05 | 1.05 - 1.06 |
| 61 | STD-max-𝛿0-13 | 0.05 | 11.3 | 1.05 | 1.05 - 1.06 |
| 62 | MAX-min-𝛿1-3 | 0.05 | 11.3 | 1.05 | 1.05 - 1.06 |
| 63 | MEAN-mean-𝛿0-8 | 0.05 | 11.1 | 1.05 | 1.05 - 1.05 |
| 64 | MEAN-std-𝛿0-12 | 0.05 | 11.1 | 1.05 | 1.05 - 1.05 |
| 65 | MAX-min-𝛿2-1 | 0.05 | 11.0 | 1.05 | 1.05 - 1.06 |
| 66 | MAX-std-𝛿1-2 | 0.05 | 10.9 | 1.05 | 1.05 - 1.05 |
| 67 | STD-mean-𝛿1-6 | 0.05 | 10.9 | 1.05 | 1.05 - 1.05 |
| 68 | MAX-std-𝛿1-7 | 0.05 | 10.8 | 1.05 | 1.05 - 1.05 |
| 69 | STD-mean-𝛿1-5 | 0.05 | 10.7 | 1.05 | 1.05 - 1.05 |
| 70 | MEAN-std-𝛿0-4 | 0.05 | 10.7 | 1.05 | 1.05 - 1.05 |
| 71 | STD-mean-𝛿2-2 | 0.05 | 10.5 | 1.05 | 1.04 - 1.05 |
| 72 | STD-max-𝛿2-4 | 0.05 | 10.4 | 1.05 | 1.05 - 1.05 |
| 73 | STD-mean-𝛿1-1 | 0.05 | 10.2 | 1.05 | 1.04 - 1.05 |
| 74 | MEAN-max-𝛿1-8 | 0.05 | 10.2 | 1.05 | 1.04 - 1.05 |
| 75 | MAX-mean-𝛿1-7 | 0.05 | 10.2 | 1.05 | 1.04 - 1.05 |
| 76 | MAX-max-𝛿2-2 | 0.05 | 10.1 | 1.05 | 1.04 - 1.05 |
| 77 | MAX-mean-𝛿2-2 | 0.04 | 10.0 | 1.05 | 1.04 - 1.05 |
| 78 | MAX-max-𝛿2-9 | 0.04 | 9.9 | 1.05 | 1.04 - 1.05 |
| 79 | STD-max-𝛿2-12 | 0.04 | 9.8 | 1.05 | 1.04 - 1.05 |
| 80 | STD-mean-𝛿1-13 | 0.04 | 9.8 | 1.05 | 1.04 - 1.05 |
| 81 | MEAN-max-𝛿1-13 | 0.04 | 9.8 | 1.04 | 1.04 - 1.05 |
| 82 | MEAN-max-𝛿1-5 | 0.04 | 9.7 | 1.04 | 1.04 - 1.05 |
| 83 | STD-mean-𝛿2-6 | 0.04 | 9.7 | 1.04 | 1.04 - 1.05 |
| 84 | STD-mean-𝛿1-11 | 0.04 | 9.7 | 1.04 | 1.04 - 1.05 |
| 85 | STD-mean-𝛿0-4 | 0.04 | 9.5 | 1.04 | 1.04 - 1.05 |
| 86 | MEAN-mean-𝛿0-1 | 0.04 | 9.5 | 1.04 | 1.04 - 1.05 |
| 87 | STD-max-𝛿0-8 | 0.04 | 9.2 | 1.04 | 1.04 - 1.05 |
| 88 | STD-std-𝛿0-5 | 0.04 | 9.1 | 1.04 | 1.04 - 1.04 |
| 89 | STD-max-𝛿1-4 | 0.04 | 8.9 | 1.04 | 1.04 - 1.04 |
| 90 | STD-std-𝛿1-11 | 0.04 | 8.9 | 1.04 | 1.04 - 1.04 |
| 91 | MAX-std-𝛿2-12 | 0.04 | 8.7 | 1.04 | 1.04 - 1.04 |
| 92 | STD-mean-𝛿2-11 | 0.04 | 8.6 | 1.04 | 1.04 - 1.04 |
| 93 | STD-mean-𝛿2-4 | 0.04 | 8.6 | 1.04 | 1.04 - 1.04 |
| 94 | STD-std-𝛿2-9 | 0.04 | 8.6 | 1.04 | 1.04 - 1.04 |
| 95 | STD-min-𝛿0-12 | 0.04 | 8.5 | 1.04 | 1.03 - 1.04 |
| 96 | MAX-min-𝛿2-5 | 0.04 | 8.4 | 1.04 | 1.03 - 1.04 |
| 97 | MEAN-mean-𝛿0-5 | 0.04 | 8.3 | 1.04 | 1.03 - 1.04 |
| 98 | STD-max-𝛿2-2 | 0.04 | 8.2 | 1.04 | 1.04 - 1.04 |
| 99 | STD-std-𝛿0-2 | 0.04 | 8.2 | 1.04 | 1.03 - 1.04 |
| 100 | MEAN-max-𝛿2-11 | 0.04 | 8.1 | 1.04 | 1.04 - 1.04 |
| 101 | STD-mean-𝛿1-12 | 0.04 | 8.1 | 1.04 | 1.03 - 1.04 |
| 102 | MEAN-max-𝛿2-13 | 0.04 | 8.0 | 1.04 | 1.03 - 1.04 |
| 103 | STD-max-𝛿2-13 | 0.03 | 7.6 | 1.03 | 1.03 - 1.04 |
| 104 | MAX-std-𝛿1-5 | 0.03 | 7.6 | 1.03 | 1.03 - 1.04 |
| 105 | STD-std-𝛿1-12 | 0.03 | 7.5 | 1.03 | 1.03 - 1.04 |
| 106 | MEAN-max-𝛿2-8 | 0.03 | 7.5 | 1.03 | 1.03 - 1.04 |
| 107 | STD-mean-𝛿2-8 | 0.03 | 7.4 | 1.03 | 1.03 - 1.04 |
| 108 | STD-max-𝛿0-6 | 0.03 | 7.4 | 1.03 | 1.03 - 1.04 |
| 109 | MAX-std-𝛿0-5 | 0.03 | 7.0 | 1.03 | 1.03 - 1.04 |
| 110 | MEAN-mean-𝛿0-11 | 0.03 | 6.9 | 1.03 | 1.03 - 1.03 |
| 111 | STD-max-𝛿1-6 | 0.03 | 6.9 | 1.03 | 1.03 - 1.03 |
| 112 | MEAN-max-𝛿1-11 | 0.03 | 6.8 | 1.03 | 1.03 - 1.03 |
| 113 | MAX-min-𝛿0-1 | 0.03 | 6.8 | 1.03 | 1.03 - 1.04 |
| 114 | MEAN-std-𝛿1-4 | 0.03 | 6.7 | 1.03 | 1.03 - 1.03 |
| 115 | MEAN-min-𝛿1-5 | 0.03 | 6.7 | 1.03 | 1.03 - 1.04 |
| 116 | STD-std-𝛿1-13 | 0.03 | 6.7 | 1.03 | 1.03 - 1.03 |
| 117 | MAX-std-𝛿2-7 | 0.03 | 6.7 | 1.03 | 1.03 - 1.03 |
| 118 | STD-max-𝛿2-6 | 0.03 | 6.6 | 1.03 | 1.03 - 1.03 |
| 119 | STD-mean-𝛿0-9 | 0.03 | 6.5 | 1.03 | 1.03 - 1.03 |
| 120 | STD-max-𝛿0-12 | 0.03 | 6.3 | 1.03 | 1.02 - 1.03 |
| 121 | MAX-min-𝛿0-11 | 0.03 | 6.2 | 1.03 | 1.02 - 1.03 |
| 122 | MEAN-mean-𝛿2-4 | 0.03 | 6.1 | 1.03 | 1.03 - 1.03 |
| 123 | MEAN-std-𝛿1-9 | 0.03 | 6.1 | 1.03 | 1.03 - 1.03 |
| 124 | MAX-mean-𝛿1-6 | 0.03 | 6.0 | 1.03 | 1.02 - 1.03 |
| 125 | MAX-max-𝛿0-1 | 0.03 | 6.0 | 1.03 | 1.02 - 1.03 |
| 126 | MEAN-max-𝛿2-2 | 0.03 | 6.0 | 1.03 | 1.03 - 1.03 |
| 127 | STD-std-𝛿0-11 | 0.03 | 5.9 | 1.03 | 1.02 - 1.03 |
| 128 | MEAN-min-𝛿2-2 | 0.03 | 5.8 | 1.03 | 1.02 - 1.03 |
| 129 | MAX-max-𝛿2-3 | 0.03 | 5.7 | 1.03 | 1.02 - 1.03 |
| 130 | MEAN-max-𝛿2-10 | 0.03 | 5.7 | 1.03 | 1.02 - 1.03 |
| 131 | STD-mean-𝛿2-7 | 0.03 | 5.7 | 1.03 | 1.02 - 1.03 |
| 132 | STD-mean-𝛿2-12 | 0.03 | 5.6 | 1.03 | 1.02 - 1.03 |
| 133 | MAX-std-𝛿0-13 | 0.03 | 5.6 | 1.03 | 1.02 - 1.03 |
| 134 | MAX-mean-𝛿0-1 | 0.02 | 5.6 | 1.03 | 1.02 - 1.03 |
| 135 | MEAN-max-𝛿2-5 | 0.02 | 5.5 | 1.03 | 1.02 - 1.03 |
| 136 | MAX-mean-𝛿1-12 | 0.02 | 5.5 | 1.03 | 1.02 - 1.03 |
| 137 | MEAN-max-𝛿0-10 | 0.02 | 5.4 | 1.02 | 1.02 - 1.03 |
| 138 | STD-max-𝛿2-7 | 0.02 | 5.3 | 1.02 | 1.02 - 1.03 |
| 139 | STD-std-𝛿0-3 | 0.02 | 5.2 | 1.02 | 1.02 - 1.03 |
| 140 | MAX-mean-𝛿1-11 | 0.02 | 5.2 | 1.02 | 1.02 - 1.03 |
| 141 | MAX-mean-𝛿1-2 | 0.02 | 5.2 | 1.02 | 1.02 - 1.03 |
| 142 | MEAN-mean-𝛿1-4 | 0.02 | 5.2 | 1.02 | 1.02 - 1.03 |
| 143 | STD-mean-𝛿2-9 | 0.02 | 5.1 | 1.02 | 1.02 - 1.03 |
| 144 | STD-max-𝛿0-7 | 0.02 | 5.1 | 1.02 | 1.02 - 1.03 |
| 145 | MAX-min-𝛿0-9 | 0.02 | 5.0 | 1.02 | 1.02 - 1.03 |
| 146 | MAX-min-𝛿1-1 | 0.02 | 5.0 | 1.02 | 1.02 - 1.03 |
| 147 | STD-max-𝛿1-7 | 0.02 | 4.9 | 1.02 | 1.02 - 1.03 |
| 148 | MAX-max-𝛿1-1 | 0.02 | 4.9 | 1.02 | 1.02 - 1.03 |
| 149 | MAX-std-𝛿2-4 | 0.02 | 4.9 | 1.02 | 1.02 - 1.03 |
| 150 | MAX-std-𝛿2-3 | 0.02 | 4.9 | 1.02 | 1.02 - 1.03 |
| 151 | MAX-max-𝛿2-5 | 0.02 | 4.9 | 1.02 | 1.02 - 1.03 |
| 152 | MEAN-mean-𝛿2-7 | 0.02 | 4.8 | 1.02 | 1.02 - 1.02 |
| 153 | MEAN-std-𝛿0-3 | 0.02 | 4.8 | 1.02 | 1.02 - 1.03 |
| 154 | STD-min-𝛿0-13 | 0.02 | 4.6 | 1.02 | 1.02 - 1.03 |
| 155 | MAX-min-𝛿2-3 | 0.02 | 4.6 | 1.02 | 1.02 - 1.03 |
| 156 | MEAN-max-𝛿1-2 | 0.02 | 4.6 | 1.02 | 1.02 - 1.02 |
| 157 | MEAN-std-𝛿2-4 | 0.02 | 4.5 | 1.02 | 1.02 - 1.02 |
| 158 | MAX-std-𝛿1-9 | 0.02 | 4.5 | 1.02 | 1.02 - 1.02 |
| 159 | STD-mean-𝛿0-3 | 0.02 | 4.5 | 1.02 | 1.02 - 1.03 |
| 160 | MEAN-max-𝛿0-13 | 0.02 | 4.4 | 1.02 | 1.02 - 1.02 |
| 161 | STD-max-𝛿2-8 | 0.02 | 4.4 | 1.02 | 1.02 - 1.02 |
| 162 | MAX-max-𝛿1-2 | 0.02 | 4.3 | 1.02 | 1.02 - 1.02 |
| 163 | MEAN-min-𝛿1-13 | 0.02 | 4.3 | 1.02 | 1.01 - 1.03 |
| 164 | MAX-mean-𝛿2-1 | 0.02 | 4.3 | 1.02 | 1.02 - 1.02 |
| 165 | MAX-std-𝛿1-1 | 0.02 | 4.3 | 1.02 | 1.01 - 1.02 |
| 166 | STD-min-𝛿1-12 | 0.02 | 4.1 | 1.02 | 1.02 - 1.02 |
| 167 | STD-max-𝛿0-4 | 0.02 | 4.1 | 1.02 | 1.01 - 1.02 |
| 168 | MAX-mean-𝛿2-6 | 0.02 | 4.0 | 1.02 | 1.01 - 1.02 |
| 169 | MEAN-mean-𝛿0-4 | 0.02 | 4.0 | 1.02 | 1.01 - 1.02 |
| 170 | MEAN-min-𝛿0-4 | 0.02 | 4.0 | 1.02 | 1.01 - 1.02 |
| 171 | STD-min-𝛿2-6 | 0.02 | 3.9 | 1.02 | 1.01 - 1.02 |
| 172 | MAX-mean-𝛿0-4 | 0.02 | 3.9 | 1.02 | 1.01 - 1.02 |
| 173 | MAX-mean-𝛿1-13 | 0.02 | 3.9 | 1.02 | 1.01 - 1.02 |
| 174 | STD-std-𝛿2-7 | 0.02 | 3.8 | 1.02 | 1.01 - 1.02 |
| 175 | STD-std-𝛿2-6 | 0.02 | 3.8 | 1.02 | 1.01 - 1.02 |
| 176 | STD-min-𝛿1-10 | 0.02 | 3.7 | 1.02 | 1.01 - 1.02 |
| 177 | STD-max-𝛿0-3 | 0.02 | 3.6 | 1.02 | 1.01 - 1.02 |
| 178 | STD-min-𝛿1-2 | 0.02 | 3.6 | 1.02 | 1.01 - 1.02 |
| 179 | MAX-min-𝛿1-9 | 0.02 | 3.5 | 1.02 | 1.01 - 1.02 |
| 180 | STD-max-𝛿0-11 | 0.02 | 3.5 | 1.02 | 1.01 - 1.02 |
| 181 | STD-min-𝛿0-8 | 0.02 | 3.4 | 1.02 | 1.01 - 1.02 |
| 182 | STD-min-𝛿1-7 | 0.02 | 3.4 | 1.02 | 1.01 - 1.02 |
| 183 | MEAN-std-𝛿2-9 | 0.02 | 3.4 | 1.02 | 1.01 - 1.02 |
| 184 | MAX-max-𝛿0-3 | 0.02 | 3.4 | 1.02 | 1.01 - 1.02 |
| 185 | STD-min-𝛿1-9 | 0.02 | 3.4 | 1.02 | 1.01 - 1.02 |
| 186 | STD-std-𝛿1-2 | 0.01 | 3.3 | 1.02 | 1.01 - 1.02 |
| 187 | MAX-std-𝛿0-4 | 0.01 | 3.1 | 1.01 | 1.01 - 1.02 |
| 188 | MEAN-std-𝛿1-12 | 0.01 | 3.0 | 1.01 | 1.01 - 1.02 |
| 189 | MEAN-min-𝛿2-5 | 0.01 | 2.9 | 1.01 | 1.01 - 1.02 |
| 190 | MAX-min-𝛿0-4 | 0.01 | 2.7 | 1.01 | 1.01 - 1.02 |
| 191 | STD-mean-𝛿1-3 | 0.01 | 2.7 | 1.01 | 1.01 - 1.02 |
| 192 | MEAN-min-𝛿2-10 | 0.01 | 2.7 | 1.01 | 1.01 - 1.02 |
| 193 | MAX-mean-𝛿0-13 | 0.01 | 2.7 | 1.01 | 1.01 - 1.02 |
| 194 | STD-std-𝛿1-10 | 0.01 | 2.6 | 1.01 | 1.01 - 1.02 |
| 195 | STD-min-𝛿2-11 | 0.01 | 2.5 | 1.01 | 1.01 - 1.01 |
| 196 | STD-std-𝛿2-2 | 0.01 | 2.5 | 1.01 | 1.01 - 1.01 |
| 197 | MEAN-max-𝛿2-3 | 0.01 | 2.4 | 1.01 | 1.01 - 1.01 |
| 198 | MEAN-max-𝛿1-7 | 0.01 | 2.4 | 1.01 | 1.01 - 1.01 |
| 199 | MEAN-min-𝛿2-12 | 0.01 | 2.4 | 1.01 | 1.01 - 1.01 |
| 200 | STD-max-𝛿1-12 | 0.01 | 2.3 | 1.01 | 1.01 - 1.01 |
| 201 | MEAN-min-𝛿0-10 | 0.01 | 2.2 | 1.01 | 1.01 - 1.01 |
| 202 | STD-max-𝛿1-11 | 0.01 | 2.2 | 1.01 | 1.01 - 1.02 |
| 203 | MEAN-min-𝛿1-12 | 0.01 | 2.2 | 1.01 | 1.01 - 1.01 |
| 204 | MAX-min-𝛿1-7 | 0.01 | 2.2 | 1.01 | 1.01 - 1.01 |
| 205 | MAX-mean-𝛿2-10 | 0.01 | 2.1 | 1.01 | 1.01 - 1.01 |
| 206 | MAX-min-𝛿2-11 | 0.01 | 2.1 | 1.01 | 1.01 - 1.01 |
| 207 | STD-std-𝛿2-10 | 0.01 | 2.0 | 1.01 | 1.01 - 1.01 |
| 208 | MEAN-max-𝛿2-7 | 0.01 | 2.0 | 1.01 | 1.01 - 1.01 |
| 209 | MAX-std-𝛿1-11 | 0.01 | 2.0 | 1.01 | 1.01 - 1.01 |
| 210 | MEAN-min-𝛿1-1 | 0.01 | 2.0 | 1.01 | 1.00 - 1.01 |
| 211 | MAX-mean-𝛿1-1 | 0.01 | 2.0 | 1.01 | 1.00 - 1.01 |
| 212 | STD-max-𝛿0-1 | 0.01 | 1.9 | 1.01 | 1.00 - 1.01 |
| 213 | STD-min-𝛿2-7 | 0.01 | 1.9 | 1.01 | 1.01 - 1.01 |
| 214 | MEAN-mean-𝛿2-9 | 0.01 | 1.8 | 1.01 | 1.01 - 1.01 |
| 215 | STD-max-𝛿2-3 | 0.01 | 1.8 | 1.01 | 1.01 - 1.01 |
| 216 | MAX-min-𝛿2-4 | 0.01 | 1.8 | 1.01 | 1.00 - 1.02 |
| 217 | MAX-mean-𝛿2-9 | 0.01 | 1.8 | 1.01 | 1.00 - 1.01 |
| 218 | MEAN-mean-𝛿1-9 | 0.01 | 1.7 | 1.01 | 1.01 - 1.01 |
| 219 | STD-mean-𝛿1-4 | 0.01 | 1.7 | 1.01 | 1.00 - 1.01 |
| 220 | STD-std-𝛿2-4 | 0.01 | 1.5 | 1.01 | 1.00 - 1.01 |
| 221 | MAX-min-𝛿1-2 | 0.01 | 1.5 | 1.01 | 1.00 - 1.01 |
| 222 | MAX-max-𝛿2-6 | 0.01 | 1.5 | 1.01 | 1.00 - 1.01 |
| 223 | MEAN-min-𝛿0-13 | 0.01 | 1.4 | 1.01 | 1.00 - 1.01 |
| 224 | MAX-mean-𝛿2-4 | 0.01 | 1.4 | 1.01 | 1.00 - 1.01 |
| 225 | MAX-min-𝛿2-6 | 0.01 | 1.3 | 1.01 | 1.00 - 1.01 |
| 226 | MEAN-mean-𝛿1-12 | 0.01 | 1.3 | 1.01 | 1.00 - 1.01 |
| 227 | MEAN-min-𝛿2-7 | 0.01 | 1.2 | 1.01 | 1.00 - 1.01 |
| 228 | MAX-mean-𝛿1-8 | 0.01 | 1.2 | 1.01 | 1.00 - 1.01 |
| 229 | STD-min-𝛿1-6 | 0.01 | 1.1 | 1.01 | 1.00 - 1.01 |
| 230 | MAX-max-𝛿1-13 | 0.00 | 1.1 | 1.01 | 1.00 - 1.01 |
| 231 | MEAN-mean-𝛿0-3 | 0.00 | 1.1 | 1.00 | 1.00 - 1.01 |
| 232 | MEAN-std-𝛿2-7 | 0.00 | 0.9 | 1.00 | 1.00 - 1.01 |
| 233 | MEAN-max-𝛿0-4 | 0.00 | 0.9 | 1.00 | 1.00 - 1.01 |
| 234 | MEAN-max-𝛿1-3 | 0.00 | 0.9 | 1.00 | 1.00 - 1.01 |
| 235 | STD-mean-𝛿1-9 | 0.00 | 0.8 | 1.00 | 1.00 - 1.01 |
| 236 | STD-mean-𝛿1-2 | 0.00 | 0.8 | 1.00 | 1.00 - 1.01 |
| 237 | MAX-std-𝛿2-6 | 0.00 | 0.7 | 1.00 | 1.00 - 1.01 |
| 238 | MEAN-std-𝛿0-13 | 0.00 | 0.6 | 1.00 | 1.00 - 1.01 |
| 239 | STD-mean-𝛿0-11 | 0.00 | 0.6 | 1.00 | 1.00 - 1.01 |
| 240 | MAX-max-𝛿2-10 | 0.00 | 0.5 | 1.00 | 1.00 - 1.01 |
| 241 | MAX-std-𝛿0-2 | 0.00 | 0.4 | 1.00 | 1.00 - 1.01 |
| 242 | MEAN-min-𝛿1-10 | 0.00 | 0.4 | 1.00 | 1.00 - 1.01 |
| 243 | MEAN-std-𝛿2-12 | 0.00 | 0.4 | 1.00 | 1.00 - 1.00 |
| 244 | STD-min-𝛿1-4 | 0.00 | 0.3 | 1.00 | 1.00 - 1.01 |
| 245 | STD-mean-𝛿2-13 | 0.00 | 0.2 | 1.00 | 1.00 - 1.00 |
| 246 | STD-min-𝛿2-3 | 0.00 | 0.1 | 1.00 | 1.00 - 1.00 |
| 247 | MAX-max-𝛿2-8 | 0.00 | 0.0 | 1.00 | 1.00 - 1.01 |
| 248 | STD-min-𝛿0-3 | -0.00 | -0.1 | 1.00 | 0.99 - 1.01 |
| 249 | STD-std-𝛿0-6 | -0.00 | -0.1 | 1.00 | 1.00 - 1.00 |
| 250 | MAX-min-𝛿2-10 | -0.00 | -0.2 | 1.00 | 1.00 - 1.00 |
| 251 | STD-min-𝛿2-5 | -0.00 | -0.2 | 1.00 | 1.00 - 1.00 |
| 252 | STD-max-𝛿2-11 | -0.00 | -0.3 | 1.00 | 1.00 - 1.00 |
| 253 | STD-std-𝛿2-1 | -0.00 | -0.3 | 1.00 | 1.00 - 1.00 |
| 254 | MAX-max-𝛿0-4 | -0.00 | -0.3 | 1.00 | 0.99 - 1.00 |
| 255 | MEAN-min-𝛿2-3 | -0.00 | -0.4 | 1.00 | 0.99 - 1.00 |
| 256 | MAX-mean-𝛿0-10 | -0.00 | -0.4 | 1.00 | 1.00 - 1.00 |
| 257 | MEAN-max-𝛿2-6 | -0.00 | -0.5 | 1.00 | 1.00 - 1.00 |
| 258 | MEAN-max-𝛿2-12 | -0.00 | -0.6 | 1.00 | 1.00 - 1.00 |
| 259 | MAX-min-𝛿0-10 | -0.00 | -0.6 | 1.00 | 0.99 - 1.00 |
| 260 | MEAN-min-𝛿0-3 | -0.00 | -0.6 | 1.00 | 0.99 - 1.00 |
| 261 | STD-min-𝛿0-2 | -0.00 | -0.7 | 1.00 | 0.99 - 1.00 |
| 262 | STD-max-𝛿1-3 | -0.00 | -0.8 | 1.00 | 0.99 - 1.00 |
| 263 | MEAN-std-𝛿0-7 | -0.00 | -0.8 | 1.00 | 0.99 - 1.00 |
| 264 | MAX-max-𝛿2-11 | -0.00 | -0.9 | 1.00 | 0.99 - 1.00 |
| 265 | MAX-min-𝛿1-5 | -0.00 | -0.9 | 1.00 | 0.99 - 1.00 |
| 266 | MEAN-mean-𝛿0-9 | -0.00 | -0.9 | 1.00 | 0.99 - 1.00 |
| 267 | STD-std-𝛿1-3 | -0.00 | -1.0 | 1.00 | 0.99 - 1.00 |
| 268 | STD-min-𝛿0-4 | -0.01 | -1.2 | 0.99 | 0.99 - 1.00 |
| 269 | STD-std-𝛿2-8 | -0.01 | -1.2 | 0.99 | 0.99 - 1.00 |
| 270 | MEAN-mean-𝛿2-12 | -0.01 | -1.2 | 0.99 | 0.99 - 1.00 |
| 271 | MAX-max-𝛿2-12 | -0.01 | -1.3 | 0.99 | 0.99 - 1.00 |
| 272 | STD-min-𝛿1-11 | -0.01 | -1.4 | 0.99 | 0.99 - 1.00 |
| 273 | MAX-max-𝛿1-3 | -0.01 | -1.5 | 0.99 | 0.99 - 1.00 |
| 274 | MAX-std-𝛿2-9 | -0.01 | -1.5 | 0.99 | 0.99 - 1.00 |
| 275 | MAX-max-𝛿0-11 | -0.01 | -1.5 | 0.99 | 0.99 - 1.00 |
| 276 | MAX-std-𝛿1-4 | -0.01 | -1.6 | 0.99 | 0.99 - 1.00 |
| 277 | STD-min-𝛿0-10 | -0.01 | -1.6 | 0.99 | 0.99 - 1.00 |
| 278 | STD-min-𝛿0-6 | -0.01 | -1.8 | 0.99 | 0.99 - 1.00 |
| 279 | STD-max-𝛿1-13 | -0.01 | -1.9 | 0.99 | 0.99 - 0.99 |
| 280 | STD-mean-𝛿2-5 | -0.01 | -2.0 | 0.99 | 0.99 - 0.99 |
| 281 | MAX-mean-𝛿2-8 | -0.01 | -2.2 | 0.99 | 0.99 - 0.99 |
| 282 | STD-max-𝛿1-8 | -0.01 | -2.2 | 0.99 | 0.99 - 0.99 |
| 283 | MEAN-mean-𝛿1-3 | -0.01 | -2.3 | 0.99 | 0.99 - 0.99 |
| 284 | STD-min-𝛿2-10 | -0.01 | -2.3 | 0.99 | 0.99 - 0.99 |
| 285 | MEAN-mean-𝛿2-5 | -0.01 | -2.4 | 0.99 | 0.99 - 0.99 |
| 286 | MEAN-max-𝛿0-3 | -0.01 | -2.5 | 0.99 | 0.99 - 0.99 |
| 287 | MEAN-std-𝛿2-10 | -0.01 | -2.6 | 0.99 | 0.99 - 0.99 |
| 288 | MEAN-mean-𝛿2-11 | -0.01 | -2.7 | 0.99 | 0.99 - 0.99 |
| 289 | MEAN-mean-𝛿2-10 | -0.01 | -2.7 | 0.99 | 0.99 - 0.99 |
| 290 | STD-std-𝛿1-8 | -0.01 | -2.8 | 0.99 | 0.98 - 0.99 |
| 291 | STD-std-𝛿0-10 | -0.01 | -2.9 | 0.99 | 0.98 - 0.99 |
| 292 | STD-min-𝛿1-5 | -0.01 | -2.9 | 0.99 | 0.98 - 0.99 |
| 293 | STD-std-𝛿2-3 | -0.01 | -3.0 | 0.99 | 0.98 - 0.99 |
| 294 | MEAN-std-𝛿1-3 | -0.01 | -3.1 | 0.99 | 0.98 - 0.99 |
| 295 | STD-mean-𝛿0-12 | -0.01 | -3.1 | 0.99 | 0.98 - 0.99 |
| 296 | MEAN-std-𝛿1-7 | -0.01 | -3.1 | 0.99 | 0.98 - 0.99 |
| 297 | STD-mean-𝛿1-7 | -0.01 | -3.2 | 0.99 | 0.98 - 0.99 |
| 298 | MAX-mean-𝛿2-11 | -0.01 | -3.2 | 0.99 | 0.98 - 0.99 |
| 299 | MAX-std-𝛿2-2 | -0.01 | -3.3 | 0.99 | 0.98 - 0.99 |
| 300 | MAX-max-𝛿0-5 | -0.01 | -3.3 | 0.99 | 0.98 - 0.99 |
| 301 | MAX-mean-𝛿2-12 | -0.01 | -3.3 | 0.99 | 0.98 - 0.99 |
| 302 | MEAN-mean-𝛿0-13 | -0.02 | -3.4 | 0.98 | 0.98 - 0.99 |
| 303 | MAX-std-𝛿1-3 | -0.02 | -3.4 | 0.98 | 0.98 - 0.99 |
| 304 | MAX-std-𝛿0-3 | -0.02 | -3.4 | 0.98 | 0.98 - 0.99 |
| 305 | STD-max-𝛿0-5 | -0.02 | -3.6 | 0.98 | 0.98 - 0.99 |
| 306 | STD-max-𝛿2-5 | -0.02 | -3.6 | 0.98 | 0.98 - 0.99 |
| 307 | MEAN-std-𝛿2-5 | -0.02 | -3.6 | 0.98 | 0.98 - 0.99 |
| 308 | MAX-std-𝛿0-6 | -0.02 | -3.7 | 0.98 | 0.98 - 0.99 |
| 309 | STD-mean-𝛿0-1 | -0.02 | -3.8 | 0.98 | 0.98 - 0.99 |
| 310 | MAX-mean-𝛿1-9 | -0.02 | -4.0 | 0.98 | 0.98 - 0.99 |
| 311 | MAX-min-𝛿1-10 | -0.02 | -4.0 | 0.98 | 0.98 - 0.99 |
| 312 | MEAN-max-𝛿0-6 | -0.02 | -4.1 | 0.98 | 0.98 - 0.98 |
| 313 | MEAN-mean-𝛿1-5 | -0.02 | -4.1 | 0.98 | 0.98 - 0.98 |
| 314 | MEAN-max-𝛿2-4 | -0.02 | -4.2 | 0.98 | 0.98 - 0.98 |
| 315 | MAX-max-𝛿1-5 | -0.02 | -4.2 | 0.98 | 0.98 - 0.99 |
| 316 | MEAN-max-𝛿1-12 | -0.02 | -4.2 | 0.98 | 0.98 - 0.98 |
| 317 | STD-std-𝛿2-13 | -0.02 | -4.3 | 0.98 | 0.98 - 0.98 |
| 318 | MEAN-mean-𝛿1-6 | -0.02 | -4.4 | 0.98 | 0.98 - 0.98 |
| 319 | MEAN-min-𝛿2-9 | -0.02 | -4.4 | 0.98 | 0.98 - 0.98 |
| 320 | MEAN-std-𝛿0-2 | -0.02 | -4.5 | 0.98 | 0.98 - 0.98 |
| 321 | STD-min-𝛿0-7 | -0.02 | -4.6 | 0.98 | 0.98 - 0.98 |
| 322 | STD-min-𝛿1-3 | -0.02 | -4.7 | 0.98 | 0.98 - 0.98 |
| 323 | STD-mean-𝛿2-10 | -0.02 | -4.7 | 0.98 | 0.98 - 0.98 |
| 324 | STD-min-𝛿2-4 | -0.02 | -4.8 | 0.98 | 0.98 - 0.98 |
| 325 | MEAN-max-𝛿0-9 | -0.02 | -4.8 | 0.98 | 0.98 - 0.98 |
| 326 | MEAN-max-𝛿2-9 | -0.02 | -4.8 | 0.98 | 0.98 - 0.98 |
| 327 | MEAN-mean-𝛿0-10 | -0.02 | -4.8 | 0.98 | 0.98 - 0.98 |
| 328 | MEAN-mean-𝛿2-8 | -0.02 | -4.9 | 0.98 | 0.98 - 0.98 |
| 329 | MEAN-min-𝛿0-9 | -0.02 | -5.1 | 0.98 | 0.98 - 0.98 |
| 330 | STD-max-𝛿2-10 | -0.02 | -5.2 | 0.98 | 0.97 - 0.98 |
| 331 | MEAN-std-𝛿2-3 | -0.02 | -5.2 | 0.98 | 0.97 - 0.98 |
| 332 | MEAN-max-𝛿1-4 | -0.02 | -5.3 | 0.98 | 0.97 - 0.98 |
| 333 | MAX-std-𝛿1-6 | -0.02 | -5.3 | 0.98 | 0.97 - 0.98 |
| 334 | MEAN-std-𝛿1-5 | -0.02 | -5.4 | 0.98 | 0.97 - 0.98 |
| 335 | STD-mean-𝛿0-8 | -0.02 | -5.4 | 0.98 | 0.97 - 0.98 |
| 336 | MEAN-mean-𝛿2-3 | -0.02 | -5.5 | 0.98 | 0.97 - 0.98 |
| 337 | MEAN-mean-𝛿2-2 | -0.02 | -5.6 | 0.98 | 0.97 - 0.98 |
| 338 | MEAN-min-𝛿0-1 | -0.03 | -5.7 | 0.97 | 0.97 - 0.98 |
| 339 | MEAN-min-𝛿2-13 | -0.03 | -5.7 | 0.97 | 0.97 - 0.98 |
| 340 | STD-max-𝛿0-10 | -0.03 | -5.8 | 0.97 | 0.97 - 0.98 |
| 341 | MEAN-min-𝛿2-8 | -0.03 | -5.8 | 0.97 | 0.97 - 0.98 |
| 342 | MEAN-mean-𝛿1-13 | -0.03 | -6.0 | 0.97 | 0.97 - 0.98 |
| 343 | MEAN-std-𝛿0-10 | -0.03 | -6.1 | 0.97 | 0.97 - 0.98 |
| 344 | MEAN-std-𝛿0-5 | -0.03 | -6.2 | 0.97 | 0.97 - 0.98 |
| 345 | STD-min-𝛿2-12 | -0.03 | -6.2 | 0.97 | 0.97 - 0.98 |
| 346 | MAX-mean-𝛿2-7 | -0.03 | -6.3 | 0.97 | 0.97 - 0.98 |
| 347 | STD-std-𝛿0-8 | -0.03 | -6.4 | 0.97 | 0.97 - 0.98 |
| 348 | MEAN-std-𝛿0-6 | -0.03 | -6.4 | 0.97 | 0.97 - 0.98 |
| 349 | MEAN-mean-𝛿1-11 | -0.03 | -6.4 | 0.97 | 0.97 - 0.97 |
| 350 | MAX-mean-𝛿2-13 | -0.03 | -6.4 | 0.97 | 0.97 - 0.98 |
| 351 | MAX-std-𝛿1-13 | -0.03 | -6.5 | 0.97 | 0.97 - 0.98 |
| 352 | MEAN-max-𝛿1-9 | -0.03 | -6.6 | 0.97 | 0.97 - 0.97 |
| 353 | MAX-min-𝛿0-5 | -0.03 | -6.6 | 0.97 | 0.97 - 0.98 |
| 354 | MAX-min-𝛿2-12 | -0.03 | -6.7 | 0.97 | 0.97 - 0.98 |
| 355 | MAX-min-𝛿0-3 | -0.03 | -6.7 | 0.97 | 0.97 - 0.97 |
| 356 | MEAN-min-𝛿1-3 | -0.03 | -6.7 | 0.97 | 0.97 - 0.97 |
| 357 | STD-mean-𝛿1-8 | -0.03 | -6.7 | 0.97 | 0.97 - 0.97 |
| 358 | STD-min-𝛿1-8 | -0.03 | -6.9 | 0.97 | 0.97 - 0.97 |
| 359 | MEAN-mean-𝛿2-6 | -0.03 | -7.0 | 0.97 | 0.97 - 0.97 |
| 360 | MAX-max-𝛿0-10 | -0.03 | -7.2 | 0.97 | 0.96 - 0.97 |
| 361 | MAX-max-𝛿1-12 | -0.03 | -7.3 | 0.97 | 0.96 - 0.97 |
| 362 | MAX-std-𝛿0-9 | -0.03 | -7.4 | 0.97 | 0.96 - 0.97 |
| 363 | STD-mean-𝛿0-13 | -0.03 | -7.5 | 0.97 | 0.96 - 0.97 |
| 364 | MEAN-std-𝛿2-11 | -0.03 | -7.5 | 0.97 | 0.97 - 0.97 |
| 365 | MAX-max-𝛿2-4 | -0.03 | -7.5 | 0.97 | 0.96 - 0.97 |
| 366 | STD-std-𝛿2-11 | -0.03 | -7.6 | 0.97 | 0.96 - 0.97 |
| 367 | MEAN-min-𝛿1-8 | -0.03 | -7.6 | 0.97 | 0.96 - 0.97 |
| 368 | MAX-max-𝛿2-7 | -0.03 | -7.6 | 0.97 | 0.96 - 0.97 |
| 369 | STD-std-𝛿1-6 | -0.04 | -7.8 | 0.97 | 0.96 - 0.97 |
| 370 | MEAN-std-𝛿2-2 | -0.04 | -8.3 | 0.96 | 0.96 - 0.97 |
| 371 | MEAN-std-𝛿1-2 | -0.04 | -8.4 | 0.96 | 0.96 - 0.97 |
| 372 | MEAN-std-𝛿1-11 | -0.04 | -8.9 | 0.96 | 0.96 - 0.96 |
| 373 | MEAN-std-𝛿2-6 | -0.04 | -8.9 | 0.96 | 0.96 - 0.96 |
| 374 | MAX-max-𝛿1-6 | -0.04 | -9.0 | 0.96 | 0.96 - 0.96 |
| 375 | MEAN-mean-𝛿1-2 | -0.04 | -9.1 | 0.96 | 0.96 - 0.96 |
| 376 | MEAN-max-𝛿0-12 | -0.04 | -9.4 | 0.96 | 0.96 - 0.96 |
| 377 | MEAN-mean-𝛿1-7 | -0.04 | -9.4 | 0.96 | 0.96 - 0.96 |
| 378 | MAX-std-𝛿1-12 | -0.04 | -9.5 | 0.96 | 0.95 - 0.96 |
| 379 | STD-min-𝛿2-9 | -0.04 | -9.6 | 0.96 | 0.95 - 0.96 |
| 380 | MAX-max-𝛿1-10 | -0.04 | -9.8 | 0.96 | 0.95 - 0.96 |
| 381 | MAX-mean-𝛿0-3 | -0.05 | -10.2 | 0.96 | 0.95 - 0.96 |
| 382 | MAX-std-𝛿0-8 | -0.05 | -10.3 | 0.96 | 0.95 - 0.96 |
| 383 | MAX-std-𝛿2-10 | -0.05 | -10.4 | 0.95 | 0.95 - 0.96 |
| 384 | MEAN-std-𝛿0-8 | -0.05 | -10.5 | 0.95 | 0.95 - 0.96 |
| 385 | MEAN-std-𝛿2-8 | -0.05 | -10.7 | 0.95 | 0.95 - 0.96 |
| 386 | MEAN-mean-𝛿2-13 | -0.05 | -10.8 | 0.95 | 0.95 - 0.95 |
| 387 | MAX-min-𝛿1-12 | -0.05 | -10.9 | 0.95 | 0.95 - 0.96 |
| 388 | MAX-mean-𝛿0-11 | -0.05 | -10.9 | 0.95 | 0.95 - 0.96 |
| 389 | MEAN-max-𝛿0-7 | -0.05 | -11.0 | 0.95 | 0.95 - 0.96 |
| 390 | MEAN-min-𝛿2-4 | -0.05 | -11.2 | 0.95 | 0.95 - 0.96 |
| 391 | MEAN-min-𝛿2-11 | -0.05 | -11.2 | 0.95 | 0.95 - 0.95 |
| 392 | MEAN-mean-𝛿1-8 | -0.05 | -11.3 | 0.95 | 0.95 - 0.95 |
| 393 | MAX-mean-𝛿1-3 | -0.05 | -11.4 | 0.95 | 0.95 - 0.95 |
| 394 | MAX-mean-𝛿1-5 | -0.05 | -11.6 | 0.95 | 0.95 - 0.95 |
| 395 | MEAN-std-𝛿1-10 | -0.05 | -12.0 | 0.95 | 0.95 - 0.95 |
| 396 | STD-mean-𝛿0-7 | -0.05 | -12.0 | 0.95 | 0.94 - 0.95 |
| 397 | STD-max-𝛿1-5 | -0.05 | -12.0 | 0.95 | 0.94 - 0.95 |
| 398 | STD-mean-𝛿0-10 | -0.05 | -12.0 | 0.95 | 0.94 - 0.95 |
| 399 | MEAN-std-𝛿1-13 | -0.05 | -12.1 | 0.95 | 0.95 - 0.95 |
| 400 | MEAN-min-𝛿0-7 | -0.05 | -12.2 | 0.95 | 0.94 - 0.95 |
| 401 | MEAN-max-𝛿0-2 | -0.05 | -12.2 | 0.95 | 0.94 - 0.95 |
| 402 | MAX-max-𝛿0-7 | -0.05 | -12.2 | 0.95 | 0.94 - 0.95 |
| 403 | MEAN-min-𝛿1-7 | -0.06 | -12.3 | 0.95 | 0.94 - 0.95 |
| 404 | MEAN-min-𝛿2-1 | -0.06 | -12.6 | 0.95 | 0.94 - 0.95 |
| 405 | STD-mean-𝛿1-10 | -0.06 | -12.9 | 0.94 | 0.94 - 0.95 |
| 406 | MAX-std-𝛿2-8 | -0.06 | -13.1 | 0.94 | 0.94 - 0.95 |
| 407 | MAX-max-𝛿0-2 | -0.06 | -13.1 | 0.94 | 0.94 - 0.95 |
| 408 | MEAN-std-𝛿2-13 | -0.06 | -13.3 | 0.94 | 0.94 - 0.94 |
| 409 | MEAN-mean-𝛿1-10 | -0.06 | -13.5 | 0.94 | 0.94 - 0.94 |
| 410 | MAX-std-𝛿0-11 | -0.06 | -13.6 | 0.94 | 0.94 - 0.94 |
| 411 | MAX-max-𝛿2-1 | -0.06 | -13.6 | 0.94 | 0.94 - 0.95 |
| 412 | MEAN-std-𝛿1-8 | -0.06 | -13.6 | 0.94 | 0.94 - 0.94 |
| 413 | STD-max-𝛿1-10 | -0.06 | -13.6 | 0.94 | 0.94 - 0.94 |
| 414 | MAX-mean-𝛿0-9 | -0.06 | -14.0 | 0.94 | 0.94 - 0.94 |
| 415 | MAX-mean-𝛿2-5 | -0.06 | -14.1 | 0.94 | 0.94 - 0.94 |
| 416 | MEAN-max-𝛿0-1 | -0.06 | -14.3 | 0.94 | 0.94 - 0.94 |
| 417 | MEAN-min-𝛿1-11 | -0.07 | -14.9 | 0.94 | 0.93 - 0.94 |
| 418 | MEAN-min-𝛿2-6 | -0.07 | -15.0 | 0.93 | 0.93 - 0.94 |
| 419 | STD-std-𝛿2-5 | -0.07 | -16.0 | 0.93 | 0.93 - 0.93 |
| 420 | MEAN-std-𝛿1-6 | -0.07 | -16.1 | 0.93 | 0.93 - 0.93 |
| 421 | MEAN-std-𝛿0-11 | -0.07 | -16.2 | 0.93 | 0.93 - 0.93 |
| 422 | MEAN-max-𝛿2-1 | -0.07 | -16.2 | 0.93 | 0.93 - 0.93 |
| 423 | MEAN-mean-𝛿0-12 | -0.07 | -16.4 | 0.93 | 0.93 - 0.93 |
| 424 | STD-mean-𝛿2-3 | -0.07 | -16.4 | 0.93 | 0.93 - 0.93 |
| 425 | MAX-min-𝛿2-13 | -0.07 | -16.5 | 0.93 | 0.93 - 0.93 |
| 426 | STD-min-𝛿0-11 | -0.08 | -16.8 | 0.93 | 0.92 - 0.93 |
| 427 | MEAN-min-𝛿0-12 | -0.08 | -16.9 | 0.93 | 0.92 - 0.93 |
| 428 | MAX-min-𝛿0-12 | -0.08 | -17.1 | 0.93 | 0.92 - 0.93 |
| 429 | MEAN-min-𝛿1-4 | -0.08 | -17.1 | 0.93 | 0.92 - 0.93 |
| 430 | MAX-std-𝛿1-10 | -0.08 | -17.4 | 0.92 | 0.92 - 0.93 |
| 431 | STD-min-𝛿1-13 | -0.08 | -17.6 | 0.92 | 0.92 - 0.93 |
| 432 | STD-mean-𝛿0-2 | -0.08 | -17.7 | 0.92 | 0.92 - 0.93 |
| 433 | MAX-min-𝛿1-8 | -0.08 | -17.8 | 0.92 | 0.92 - 0.93 |
| 434 | MAX-max-𝛿0-12 | -0.08 | -17.8 | 0.92 | 0.92 - 0.93 |
| 435 | MAX-std-𝛿2-11 | -0.08 | -18.2 | 0.92 | 0.92 - 0.92 |
| 436 | MAX-max-𝛿1-11 | -0.08 | -18.3 | 0.92 | 0.92 - 0.93 |
| 437 | MEAN-mean-𝛿0-2 | -0.08 | -18.3 | 0.92 | 0.92 - 0.92 |
| 438 | MAX-mean-𝛿0-7 | -0.09 | -19.1 | 0.92 | 0.91 - 0.92 |
| 439 | MEAN-mean-𝛿0-6 | -0.09 | -19.4 | 0.92 | 0.91 - 0.92 |
| 440 | MEAN-max-𝛿1-1 | -0.09 | -19.6 | 0.92 | 0.91 - 0.92 |
| 441 | MEAN-min-𝛿1-6 | -0.09 | -19.9 | 0.91 | 0.91 - 0.92 |
| 442 | MEAN-min-𝛿0-2 | -0.09 | -19.9 | 0.91 | 0.91 - 0.92 |
| 443 | MAX-std-𝛿1-8 | -0.09 | -20.1 | 0.91 | 0.91 - 0.92 |
| 444 | MAX-min-𝛿1-6 | -0.09 | -20.3 | 0.91 | 0.91 - 0.92 |
| 445 | MAX-mean-𝛿0-12 | -0.09 | -20.6 | 0.91 | 0.91 - 0.92 |
| 446 | MEAN-mean-𝛿0-7 | -0.09 | -21.0 | 0.91 | 0.91 - 0.91 |
| 447 | MAX-std-𝛿2-1 | -0.09 | -21.1 | 0.91 | 0.91 - 0.91 |
| 448 | MAX-std-𝛿0-10 | -0.10 | -21.3 | 0.91 | 0.91 - 0.91 |
| 449 | MAX-mean-𝛿2-3 | -0.10 | -21.4 | 0.91 | 0.90 - 0.91 |
| 450 | MAX-mean-𝛿0-2 | -0.10 | -21.5 | 0.91 | 0.90 - 0.91 |
| 451 | MAX-min-𝛿2-2 | -0.10 | -22.1 | 0.91 | 0.90 - 0.91 |
| 452 | MAX-min-𝛿0-2 | -0.10 | -22.3 | 0.90 | 0.90 - 0.91 |
| 453 | MAX-min-𝛿2-9 | -0.10 | -22.3 | 0.90 | 0.90 - 0.91 |
| 454 | STD-min-𝛿2-13 | -0.10 | -22.4 | 0.90 | 0.90 - 0.91 |
| 455 | STD-min-𝛿2-2 | -0.10 | -22.4 | 0.90 | 0.90 - 0.91 |
| 456 | MAX-max-𝛿0-6 | -0.10 | -22.4 | 0.90 | 0.90 - 0.91 |
| 457 | MAX-min-𝛿0-6 | -0.11 | -24.6 | 0.90 | 0.89 - 0.90 |
| 458 | MAX-min-𝛿2-8 | -0.11 | -24.9 | 0.89 | 0.89 - 0.90 |
| 459 | MAX-mean-𝛿0-6 | -0.12 | -26.5 | 0.89 | 0.88 - 0.89 |
| 460 | MAX-std-𝛿2-13 | -0.13 | -29.0 | 0.88 | 0.87 - 0.88 |
| 461 | MEAN-min-𝛿0-6 | -0.13 | -29.0 | 0.88 | 0.87 - 0.88 |
| 462 | MAX-std-𝛿2-5 | -0.13 | -29.6 | 0.88 | 0.87 - 0.88 |
| 463 | MAX-min-𝛿1-4 | -0.14 | -30.5 | 0.87 | 0.87 - 0.88 |
| 464 | MAX-mean-𝛿1-10 | -0.14 | -31.8 | 0.87 | 0.86 - 0.87 |
| 465 | STD-min-𝛿2-8 | -0.15 | -32.3 | 0.86 | 0.86 - 0.87 |
| 466 | MAX-min-𝛿1-11 | -0.15 | -32.4 | 0.86 | 0.86 - 0.87 |
| 467 | MAX-min-𝛿0-7 | -0.15 | -32.5 | 0.86 | 0.86 - 0.87 |
| 468 | MAX-min-𝛿1-13 | -0.15 | -32.8 | 0.86 | 0.86 - 0.87 |
